# Climatic and topographic changes since the Miocene influenced the diversification and biogeography of the tent tortoise (*Psammobates tentorius*) species complex in Southern Africa

**DOI:** 10.1186/s12862-020-01717-1

**Published:** 2020-11-13

**Authors:** Zhongning Zhao, Neil Heideman, Phillip Bester, Adriaan Jordaan, Margaretha D. Hofmeyr

**Affiliations:** 1 Department of Zoology and Entomology, University of the Free State, Biology Building B19, 205 Nelson Mandela Dr, Park West, Bloemfontein, South Africa; 2 Department of Virology, University of the Free State and National Health Laboratory Service (NHLS), Bloemfontein, South Africa; 3 Department of Biodiversity and Conservation Biology, Chelonian Biodiversity and Conservation, University of the Western Cape, Bellville, South Africa

**Keywords:** Cladogenesis, Evolutionary driver, Miocene cooling, Refugia retracting, Biogeography, Hidden diversity

## Abstract

**Background:**

Climatic and topographic changes function as key drivers in shaping genetic structure and cladogenic radiation in many organisms. Southern Africa has an exceptionally diverse tortoise fauna, harbouring one-third of the world’s tortoise genera. The distribution of *Psammobates tentorius* (Kuhl, 1820) covers two of the 25 biodiversity hotspots in the world, the Succulent Karoo and Cape Floristic Region. The highly diverged *P. tentorius* represents an excellent model species for exploring biogeographic and radiation patterns of reptiles in Southern Africa.

**Results:**

We investigated genetic structure and radiation patterns against temporal and spatial dimensions since the Miocene in the *Psammobates tentorius* species complex, using multiple types of DNA markers and niche modelling analyses. Cladogenesis in *P. tentorius* started in the late Miocene (11.63–5.33 Ma) when populations dispersed from north to south to form two geographically isolated groups. The northern group diverged into a clade north of the Orange River (OR), followed by the splitting of the group south of the OR into a western and an interior clade. The latter divergence corresponded to the intensification of the cold Benguela current, which caused western aridification and rainfall seasonality. In the south, tectonic uplift and subsequent exhumation, together with climatic fluctuations seemed responsible for radiations among the four southern clades since the late Miocene. We found that each clade occurred in a habitat shaped by different climatic parameters, and that the niches differed substantially among the clades of the northern group but were similar among clades of the southern group.

**Conclusion:**

Climatic shifts, and biome and geographic changes were possibly the three major driving forces shaping cladogenesis and genetic structure in Southern African tortoise species. Our results revealed that the cladogenesis of the *P. tentorius* species complex was probably shaped by environmental cooling, biome shifts and topographic uplift in Southern Africa since the late Miocene. The Last Glacial Maximum (LGM) may have impacted the distribution of *P. tentorius* substantially. We found the taxonomic diversify of the *P. tentorius* species complex to be highest in the Greater Cape Floristic Region. All seven clades discovered warrant conservation attention, particularly Ptt-B–Ptr, Ptt-A and Pv-A.

## Background

Climate fluctuations have often been invoked to explain floral and faunal diversification on a global [1–3] as well as a regional scale, e.g., in Southern Africa [4, 5]. Global cooling after the early Eocene Climatic Optimum led to the formation of the Antarctic ice-sheet by the early Oligocene, where after temperatures increased and remained high from the late Oligocene to the mid-Miocene Climatic Optimum, 17–15 Ma [6]. Subsequently, decreasing temperatures re-established the Antarctic ice-sheet by 10 Ma and established the Arctic ice-sheet by 3.2 Ma [6]. Apart from global cooling, Southern Africa became progressively more arid since the Oligocene [7], and the development of the Benguela Current 14–10 Ma [8, 9] increased western aridity and established seasonal rainfall patterns over Southern Africa by the late Miocene [4, 8].

Besides climate, landscape evolution also contributed to genetic diversification of Southern Africa’s fauna and flora [5], with the Cape Fold Mountains (CFMs), Great Escarpment (GE), and Orange River (OR) identified as potential geographic barriers to gene exchange in some taxa [10–12]. The CFMs were already formed before rifting of Gondwanaland in the lower Cretaceous [13], but differential uplift and exhumation in the last 30 million years [14] may have contributed to the current relief, and influenced distribution corridors. The GE runs 50–200 km inland from the coastline and separates the coastal plains from an elevated plateau more than 1000 m above sea level [13, 15]. Many researchers believe that the GE represents a passive, erosional remnant of the continental margin after the break-up of Gondwanaland, with its current topography dating to the end of the Cretaceous ([15] and references therein). In contrast, some geomorphological and thermochronological studies advocate that upward flexures 30 Ma [7] or 20 to 5 Ma [16], followed by erosion, established or altered the topography of the GE. Uplift events changed the course of fluvial systems [17], which can also affect genetic exchange among populations [11]. The current course of the OR represents the confluence of two paleo river systems [18]. The paleo OR (Kalahari River) drained most of Namibia and southern Botswana since the Cretaceous while the Karoo River (Orange and Vaal Rivers) drained most of South Africa and had its mouth further south at the current Olifants River [18]. The Kalahari River captured the upper courses of the Karoo River by the early Cenozoic [18, 19].

Floral development in Southern Africa has been studied extensively, with strong evidence that late Neogene climatic and geomorphic evolution (due to tectonic events) shaped the development of most current biomes [20, 21]. Reptile diversity is comparatively high in Southern Africa [22–24] and genetic diversification in several taxa has been linked to climatic change, particularly during the Pliocene–Pleistocene period, although some cladogenic events date back to the Miocene [5]. In addition, landscape features have been postulated as having demarcated phylogeographic clades in some reptile taxa [25–27].

The shifting in climate and topographical features due to historical climate and geographical events plays important roles in driving diversification and changing of genetic structure of organisms [28]. The Pleistocene climatic oscillations (2.58–0.01 Ma) in the Quaternary Epoch [29] proved to be the fundamental driver that shaped distribution patterns and cladogenesis in many organismal groups [28, 30, 31], and therefore facilitated their genetic divergence and speciation [32–34]. During the cold phase, glaciations resulted in migrations and shrinking of populations into different subpopulations, thus functioning as major drivers subdividing populations. During warm periods, the glaciers retracted, leading to recolonization, dispersal, and population expansion [32–34]. If under such climatic changes geographical events took place simultaneously, diversification and speciation would have been further reinforced and accelerated under their combined effect.

Southern Africa has an exceptionally diverse tortoise fauna, harbouring one-third of the world’s tortoise genera [35], six genera with 14 species occurring in this region [35]. Cladogenesis of Southern African tortoises at the genus level occurred mainly in the Eocene, with most species diverging between the Oligocene and mid-Miocene [36]. The latter study could not resolve relationships among the three *Psammobates* species, possibly due to rapid speciation between the late Oligocene and early Miocene. The study also showed substantial divergence within *Psammobates tentorius*, indicating the possible existence of hidden species diversity or possibly the results of localized extinctions. However, a recent tortoise phylogenomic study based on the complete mtDNA genome revealed that the genus *Psammobates* branched-off from *Stigmochelys* in the late Oligocene, and that the split between *Psammobates geometricus* and *Psammobates oculifer* was estimated to have started in the early Miocene.

**Fig. 1:**
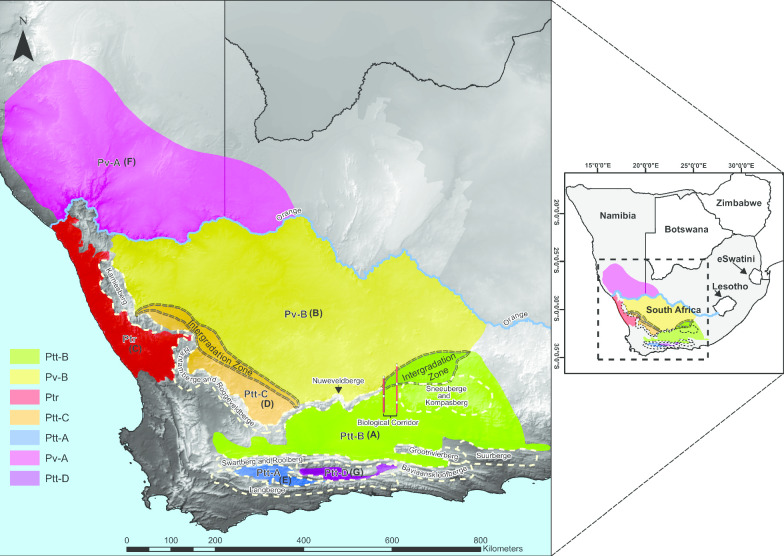
Map showing the geographic distribution of the seven clades throughout their range. The “Biological Corridor” at the intergradation zone between Ptt-B and Pv-B is shown. The Orange River between South Africa and Namibia is indicated with a blue line. Major mountain barriers are indicated. The seven geographic regions retrieved from the SAMOVA analysis are indicated as A–G. Map originally developed by Z. Zhao

Regarding nomenclature, Hewitt [37, 38] described many species and subspecies based on regional character colour variations. Loveridge and Williams [39] recognised only three subspecies, *Psammobates tentorius tentorius* (Bell, 1828), *Psammobates tentorius verroxii* (Smith, 1839) and *Psammobates tentorius trimeni* (Boulenger, 1886). This was also subsequently supported by Branch [22] and Bates et al. [24]. There are, however, still unresolved taxonomic complexities in some populations, particularly in *P. t. verroxii*, where the levels of polymorphism are the highest, warranting a re-evaluation of some of its synonymised taxa [24, 35]. There are substantial distribution range overlaps among the three subspecies, with seemingly hybrid individuals found in the intergradation zones, though many are believed to be misidentifications [24, 35]. In a subsequent study, Zhao et al. [40] used six mtDNA markers, one nDNA marker, and multiple species delimitation methods, to investigate the phylogenetic structure of *P. tentorius*. The mtDNA phylogeny distinguished seven monophyletic clades with strong support, with each clade occurring in a distinct geographic region (Fig. 1). These findings placed four clades in the current *P. t. tentorius* group and two clades in the current *P. t. verroxii* group but the latter group was however found not to be monophyletic. A single clade was assigned to the current *P. t. trimeni* group. In this study, we will label these clades as follows: the four clades in *P. t. tentorius* group: Ptt-A, Ptt-B, Ptt-C and Ptt-D; the two clades in *P. t. verroxii* group: Pv-A and Pv-B, and the single clade in the *P. t. trimeni*: Ptr.

**Fig. 2:**
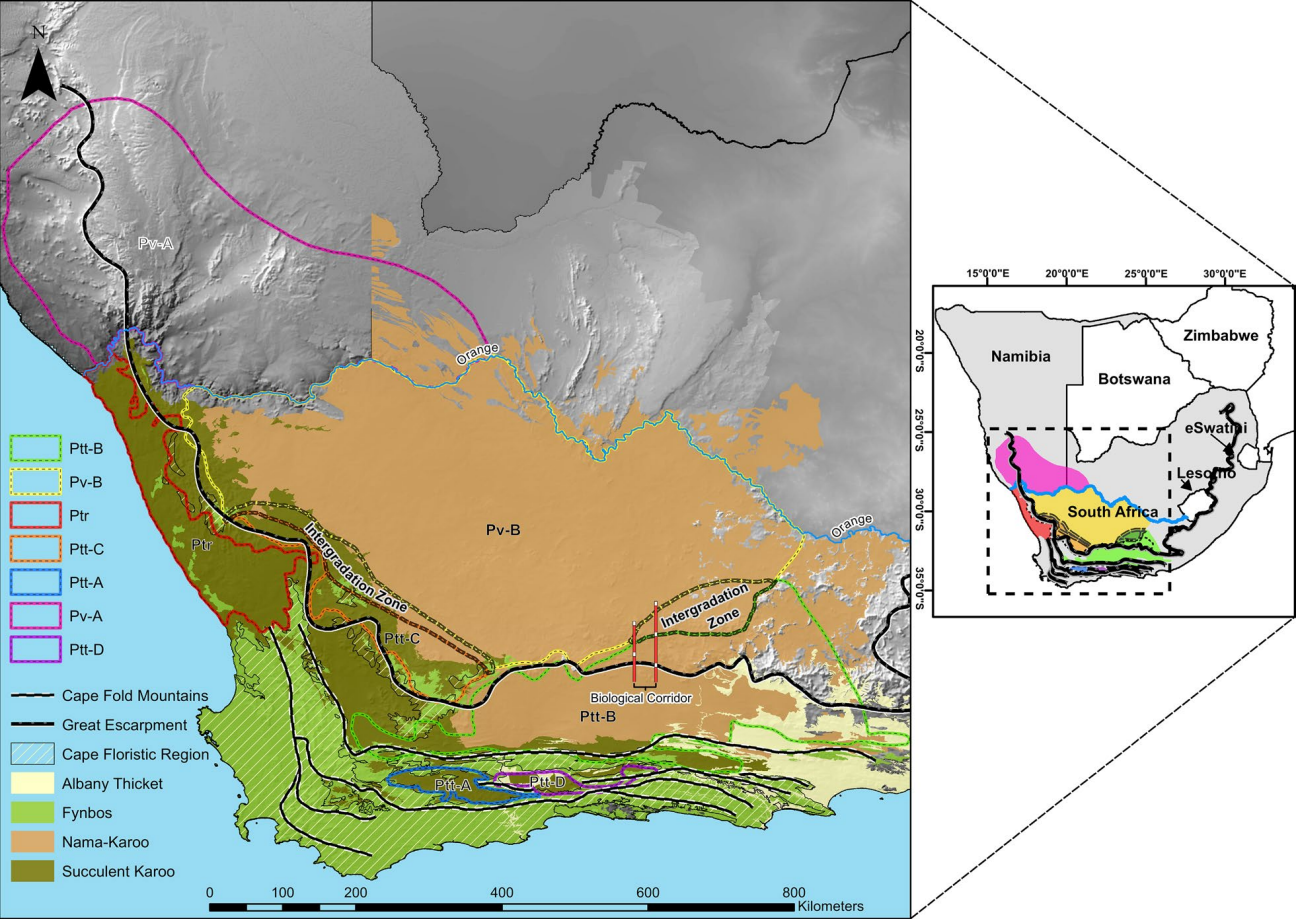
Map showing the geographic distribution of the seven clades throughout their range, together with the Cape Fold Mountains, the Great Escarpment, and the Cape Floristic Region. The ranges of the four biomes: Albany Thicket, Fynbos, Nama Karoo, and Succulent Karoo were retrieved from the biome layer of Mucina and Rutherford [175]; the layer did not include Namibia. Map originally developed by Z. Zhao

The distribution of *P. tentorius* covers two of the 25 biodiversity hotspots in the world (Fig. 2), the Succulent Karoo and Cape Floristic Region (CFR); the latter consisting mainly of the vegetation type referred to as Fynbos [41]. In recent years, botanists combined the CFR with the western winter-rainfall region of Southern Africa (mostly Succulent Karoo) below and above the western GE, to constitute a region of floral richness and endemism, the Greater Cape Floristic Region (GCFR) [42]. This region also has exceptionally high reptile diversity and endemism [5]. Apart from occurring in the GCFR, the distribution of *P. tentorius* also extends into the Nama Karoo biome of central South Africa and southern Namibia, which has the lowest floral diversity of all the biomes on the subcontinent [4]. The southeasternmost range of *P. tentorius* penetrates the westernmost distribution of Albany Thicket, a tropical biome with low floral diversity in the western region [43].

In view of its extensive genetic diversity, low dispersal ability caused by a long lifespan, slow movement and occurrence in regions of high and low floral richness and endemism, *P. tentorius* represents an excellent model species for exploring biogeographic and cladogenic patterns of reptiles in Southern Africa. Ecologically, *P. tentorius* only occurs in arid semi-desert areas, never approaching water bodies and cannot swim ([22] and Z. Zhao, pers. obs). Besides that, *P. tentorius* does not occur on slopes or in mountainous areas, but only in flat areas (Z. Zhao, pers. obs.), usually with a loose sandy base [22]. The reasons for it not occurring in mountainous or sloped areas is possibly due to its morphology. The highly domed carapace (particularly in females) may result in challenges with respect to balance, limiting its ability to navigate slopes and climb mountains. Mountains and rivers are hypothesized to be significant barriers, which restrict the movement of *P. tentorius*.

To deeply understand the diversification pattern of the *P. tentorius* species complex, besides molecular aspect, it is also crucial to address the relationship between ecological processes and speciation. In the recent years, ecological studies have become important aspect in species delimitation [44], especially for studies focusing on prediction of the suitable niches, since the variation in suitability of the habitats leads to the occurrence of different species. Various ecological niche modelling (ENM) approaches have risen to prominence in the past decade that enable the answering of deeper evolutionary questions about niche evolution, speciation and the accumulation of ecological diversity within clades [45–48]. These ENM methods also became essential for identifying suitable habitat and for predicting habitat suitability temporal-spatially, making them critically important to investigations in evolutionary and conservation biology [49].

In this study, we investigated whether the microsatellite DNA based genetic structure derived for the *P. tentorius* species complex corroborated the phylogeny retrieved using mtDNA and nDNA sequence data. Our main goal with this study was to address the cladogenic history of this highly polymorphic species complex, identify the potential drivers, and to try and establish how they shaped its diversification patterns and geographic distribution against temporal and spatial dimensions. We hypothesized that climate fluctuations and associated habitat changes over the Neogene and Quaternary influenced cladogenesis and occurrence in *P. tentorius*. Besides these, the physical barriers, in the form of either unsuitable climate or topography, possibly obstructed dispersal routes, leading to isolation and allopatric diversification. We also postulated that the diversification rate and taxon diversify would be higher in the Fynbos and Succulent Karoo vegetation of the GCFR than in the other biomes. From an ecological perspective, we hypothesized that the different candidate species occurred in distinct ecological niches, and that the suitability of the niches of each one varied with time. Finally, we postulated that environmental and climatic variables drove range changes against temporal and spatial dimensions. To test these hypotheses, we calibrated a dated phylogeny to evaluate if the timing of cladogenic events corresponded to specific climatic and/or landscape evolutionary events. We used the character dependency analyses and habitat reconstruction analyses to assess how the geographic barriers and biome shifts possibly influenced the speciation pattern of the *P. tentorius* species complex. We also used ENM techniques to evaluate ecological niches across the seven candidate species against temporal and spatial dimensions. Lastly, the diversification rate and ENM analyses were performed to evaluate its conservation status and to predict likely future trends.

## Results

### Genetic structure and phylogeny

**Fig. 3:**
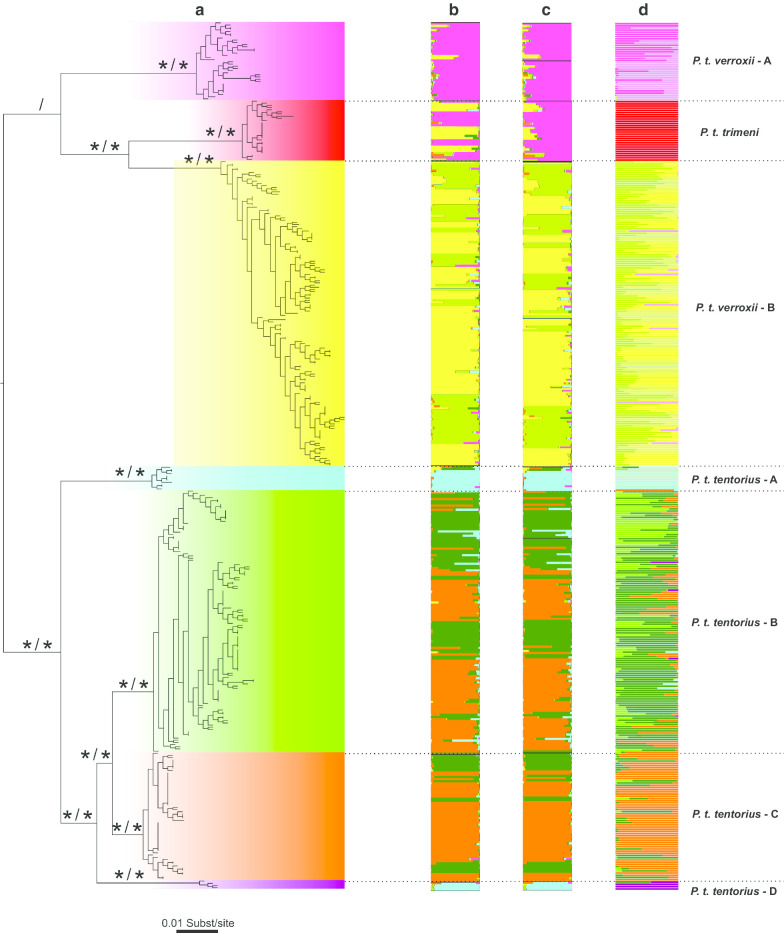
Phylogenetic tree retrieved from Bayesian inference (BI) and Maximum likelihood (ML) analyses with the mtDNA + nDNA dataset. **a** The tree topology was generated from the ML analysis. Nodes with “ *” indicate strong support (BP > 70, PP > 0.95). The clustering results from the STRUCTURE analyses (K = 6) based on **b** sub-populations, and **c** on clades. **d** DAPC analysis with 14 microsatellite DNA loci are shown to the right of the tree. “ /” indicates nodes weakly supported by both BI and ML analyses. The currently advocated “three-subspecies assumption” scheme is indicated on the right side

**Fig. 4:**
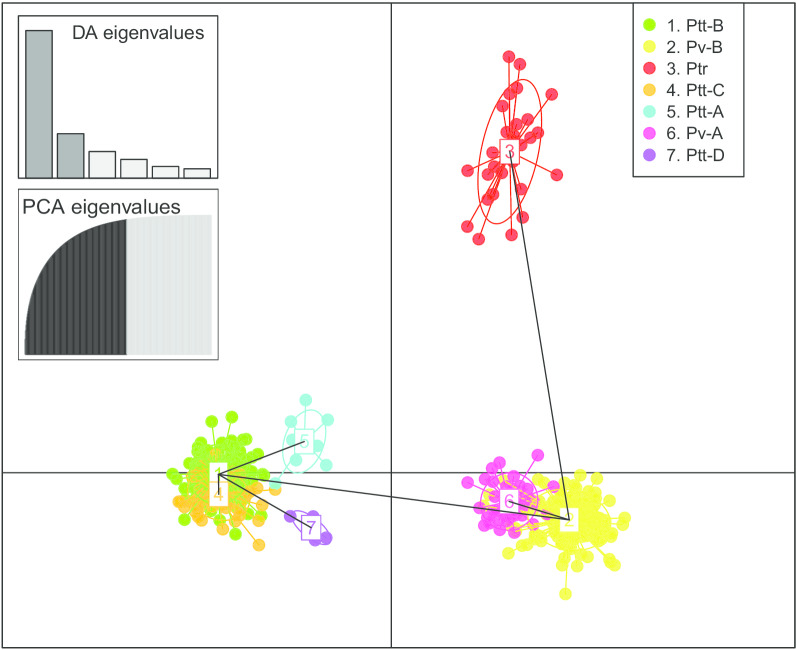
The scatterplot retrieved from the DAPC analysis (K = 7 for the best BIC score) among the seven groups corroborated the seven phylogenetic clades. The accumulative eigenvalues for the discriminant functions and principal components were shown

STRUCTURE output was interpreted using the *ΔK* method described by Evanno et al*.* [50]. The highest *ΔK* was found at K = 6 with the second highest at K = 5 (Additional file 1: Figure S1). The optimal clustering scheme under K = 6 corresponded to the five putative species for both datasets partitioned by clades and subclades (Fig. 3a–c). The difference between Ptr and Pv-A was, however, not clear (Fig. 3b–c). Furthermore, the STRUCTURE results failed to distinguish between Ptt-B and Ptt-C, and Ptt-A and Ptt-D. The differences between Ptr and Pv-A were nonetheless significant for scenarios K = 7 to K = 11 (results not shown). The DAPC membership cluster analysis, however, retrieved seven significant clusters as optimal membership clustering scheme (Fig. 3d), which perfectly corroborated the seven mtDNA clades retrieved in the previous study. The BIC criterion based DAPC analysis revealed seven clusters (K = 7) as the optimal clustering scheme (Additional file 1: Figure S2a). At K = 7, the seven clusters correspond explicitly to the seven clades in phylogenetic trees. From the 300 PCs in total used during DAPC analysis, our Cross-validation test indicated that, at approximately 100 PCs, the proportion of successful outcome prediction was highest (Additional file 1: Figure S2b). We therefore retained 100 PCs as the optimal number of PCs to construct the DAPC scatterplot. Overall, the scatterplot revealed seven distinct clusters that match the seven clades, though there was a considerable overlap between Ptt-B and Ptt-C (Fig. 4).

Regarding at sequence polymorphism (without outgroups, see Additional file 2: Table S1), the most informative genes were *Cyt-b* (fragment length: 693 bp, number of parsimony informative sites: 120, proportion of parsimony informative site: 17.32%) and *ND4* (fragment length: 678 bp, number of parsimony informative sites: 79, proportion of parsimony informative site: 11.65%), whilst, the least informative gene was *PRLR* (fragment length: 525 bp, number of parsimony informative sites: 15, proportion of parsimony informative site: 2.86%).

Both ML and Bayesian Inference (BI) analysis results for the combined dataset (mtDNA + nDNA) generated a tree topology similar to the mtDNA based phylogenetic tree of [40], except for some differences in branch lengths (Fig. 3a). The phylogenetic trees of this study retrieved two main branches, namely, northern and southern group. The northern group consisted of Pv-B, Ptr and Pv-A, all of these clades occurring in northern side of the GE (Figs. 1,2). Pv-B occurs south of the OR and above the GE, Ptr occurs in west coast, and Pv-A occurs north of the OR. Pv-B and Ptr formed a sister group. In the southern group, Ptt-B, Ptt-C, Ptt-A and Ptt-D all occurs in distinct geographic regions, and these regions are separated by mountain barriers (see Fig. 1). The phylogenetic relationships among the four clade (Ptt-B + Ptt-C) + Ptt-D) + Ptt-A were well supported by both ML and BI analyses (BP > 70, PP > 0.95). Ptt-B occurs between GE and the Swartberg Mountain, except for a population in the Nuweveldberge and Sneeuberge on the northern side of GE, Ptt-C occurs eastern side of the Hantamberge and Roggeveldberge, also on the northern side of the GE, while Ptt-A and Ptt-D are both present in the Little Karoo on southern side of Swartberg Mountain (Fig. 1). Overall, the different nodes were generally well supported, (BP > 70, PP > 0.95), except the node between (Pv-B + Ptr) and Pv-A (BP < 70, PP < 0.95). The mtDNA + nDNA tree topology also revealed low support values for node Pv-A + (Pv-B + Ptr) (BP < 70, PP < 0.95). These findings are congruent with the results reported in [37].

The taxon diversity in the Fynbos and Succulent Karoo vegetation of the GCFR (six clades in total: Ptt-B–Ptt-A– and Ptt-D) was higher than in the Nama Karoo (three clades in total: Ptt-B, Pv-B and Pv-A) and the marginally occurring Albany Thicket (only Ptt-B), see Fig. 2 for the details.

Our Migrate analysis results (see Additional file 2: Table S2) revealed overall lower levels of geneflow among Ptt-B, Ptt-C, Ptt-A and Ptt-D of the southern group. Between Ptt-A and Ptt-B it was M_5–>1_ = 4.348), between Ptt-D and Ptt-B, M_7–>1_ = 9.046, between Ptt-D and Ptt-A, M_7–>5_ = 10.614, between Ptt-A and Ptt-C, M_5–>4_ = 20.282, between Ptt-D and Ptt-C, M_7–>4_ = 20.568). The exception was the gene flow level between Ptt-B and Ptt-C which was comparatively high at M_4–>1_ = 54.900. Gene flow among the three clades (Pv-B, Ptr and Pv-A) of the northern group was also comparatively low, between Pv-A and Pv-B, M_6–>2_ = 23.986, between Ptr and Pv-B, M_3–>2_ = 20.531, and between Ptr and Pv-A, M_3–>6_ = 19.78. On the other hand, gene flow levels in the intergradation zones between Ptt-B and Pv-B, and between Pv-B and Ptt-C were also comparatively high, but especially between Ptt-B and Pv-B, M_1–>2_ = 128.374, compared to Pv-B and Ptt-C, M_2–>4_ = 55.752.

### Divergence dating analyses

**Fig. 5:**
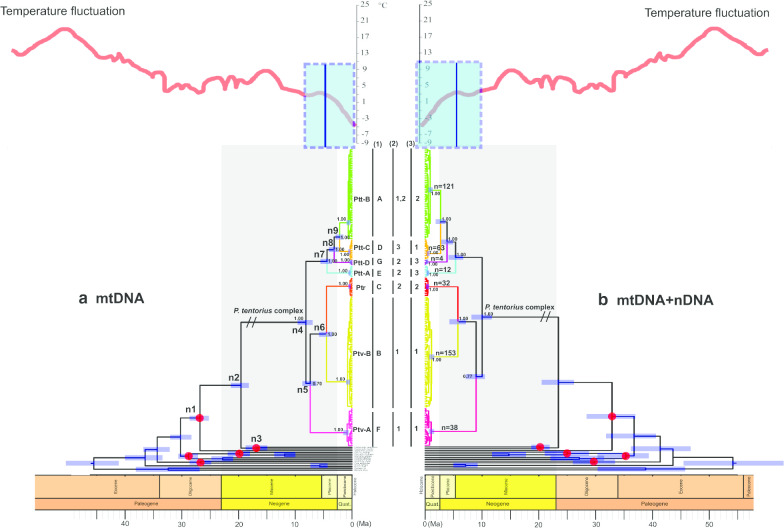
The chronograms from the concatenated dataset generated from the BEAST Bayesian calibration dating analyses with a background temperature fluctuation diagram ( modified from Zachos et al. [6, 58]). **a** The mtDNA based dating results, **b** the mtDNA + nDNA based dating results. The interval of the diversification of *P. tentorius* highlighted in blue colour represents the re-establishment of major ice sheets due to cooling. The red spots symbolize the five calibration nodes from the literature used for the calibration dating analyses. Three grouping schemes: (1) The seven geographic regions defined by the SAMOVA analysis are indicated as “A–G”. The distribution of the seven clades, (2) the biome distribution, “1” represents the Nama Karoo, “2” = Succulent Karoo and “3” = Fynbos, (3) the regions separated by the critical barriers, “1” = north of the Great Escarpment (GE), “2” = region between GE and Swartberg Mountain (SM), “3” = south of the SM. The sample size of each clade was also given. Nodes n1–n9 represent the divergence events that the cladogenic diversification of the genus *Psammobates* involved. The posterior probabilities are indicated at each node in the *P. tentorius* species complex

When comparing the dating results between the gene tree and species tree (Additional file 1: Figure S3) for both the mtDNA dataset (Fig. 5a) and the combined dataset (Fig. 5b), we found that the dating results were nearly identical (in terms of tree topology). Also, the tree topologies of the mtDNA dataset and the combined dataset showed no conflicts for both the gene trees and species trees. For both the gene tree and species tree, the combined dataset exhibited older age estimates, possibly due to the much slower evolutionary rate in the nDNA loci. Older age estimations by nDNA genes have also been found in other studies on true seals [51] and snakes [52].

The total evidence chronogram (based on the StarBEAST species tree of the mtDNA + nDNA dataset) revealed that the first divergence that split *Stigmochelys pardalis* (Bell, 1828) from the genus *Psammobates* occurred approximately 33.28 Ma (ranging from 37.9–28.99 Ma, at node n1, see Fig. 5b and Additional file 1: Table S3) in the Eocene–Oligocene. *Psammobates tentorius* branched off from the sister group comprising *P. geometricus* + *P. oculifer* approximately 23.1 Ma (ranging from 26.01–18.07 Ma, at node n2) at the Oligocene–Miocene boundary. The divergence between *P. geometricus* and *P. oculifer* dated back to the early Miocene at approximately 20.12 Ma (ranging from 21.57–18.07 Ma, at node n3). The diversification of the *P. tentorius* species complex (node n4) started in the late Miocene, approximately 10 Ma (ranging from 11.79–8.2 Ma), before the late Miocene cooling, and after the mid-Miocene Climatic Optimum. Soon after the start of the divergence, at approximately 9.14 Ma (ranging from 10.52–6.97 Ma), Pv-A split off from Pv-B + Ptr (node n5). At approximately 5.7 Ma (ranging from 7.26–4.26 Ma), Pv-B and Ptr diverged (node n6) and more or less simultaneously, Ptt-A segregated from (Ptt-B + Ptt-C) + Ptt-D approximately 5 Ma (ranging from 6.17–3.77 Ma, at node n7). The splitting of Ptt-D from (Ptt-B + Ptt-C) occurred at approximately 3.5 Ma (ranging from 4.56–2.62 Ma, at node n8) whereas Ptt-B and Ptt-C diverged at approximately 2.5 Ma (ranging from 3.38–1.63 Ma, at node n9). The rest of the cladogenic events within clades occurred within the Pleistocene epoch.

### Spatial analysis of molecular variance (SAMOVA) results and habitat reconstruction

The SAMOVA analysis (when K = 7) retrieved seven clusters that corroborated the seven phylogenetic clades (based on mtDNA and combined datasets), with strong support (fixation indices F_SC_ = 0.11, F_ST_ = 0.82 and F_CT_ = 0.8). In total, 80.04% of the total variance occurred among groups (Va: df = 6, sum of squares = 14,555.33, variance = 114.24), 17.73% of the variance came from within populations (Vc: df = 112, sum of squares = 2834.23, variance = 25.31) and only 2.23% of the variance came from within groups (Vb: df = 46, sum of squares = 1583.72, variance = 3.18). Significance tests with 1023 permutations confirm this strong genetic structure against spatial dimensions (Vc and F_ST_, *p* < 0.0001; Vb and F_SC_, *p* < 0.0001 and Va and F_CT_, *p* < 0.0001).

**Fig. 6:**
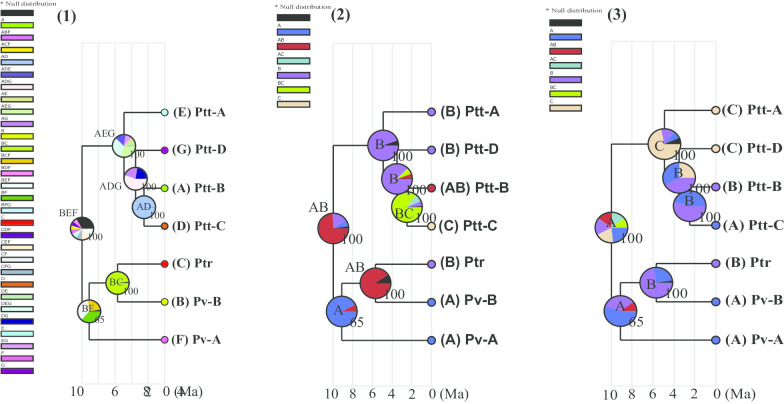
Three habitat reconstruction analysis results using the BioGeoBEARS package: (1) the reconstruction based on the seven geographic regions (A–G) defined by the SAMOVA results, (2) reconstruction based on biomes, A: Nama Karoo, B: Succulent Karoo and C: Fynbos, (3) based on the regions separated by the critical barriers, A: north of the Great Escarpment (GE), B: region between GE and Swartberg Mountain (SM), and C: south of the SM. The estimated possible ancestral area with the tree topology support values are given on the pie charts for each of the analyses

The BioGeoBEARS model test suggested “DIVALIKE + J” as the best model with the highest AICc weight score (see Additional file 1: Table S4) and a significant *p*-value in the LRT test (*p* < 0.0001) for all three analyses. The analysis based on the seven geographic regions suggested that all cladogenic events were caused by vicariance, but that the cladogenic events at nodes n4, n5 and n7 were also influenced by dispersal (Table 1 and Fig. 6). Our results revealed that both dispersal and vicariance events caused bifurcation between the two major branches, the northern (Pv-B + Ptr) + Pv-A) and southern (Ptt-B + Ptt-C) + Ptt-D) + Ptt-A). Pv-A and its habitat F was isolated from that of Pv-B + PTR, and Ptt-A separated from (Ptt-B + Ptt-C) + Ptt-D). The separation between Pv-B and Ptr, the branching off of Ptt-D from Ptt-B + Ptt-C, and the split between Ptt-B and Ptt-C were all driven by vicariance events. The diversification originated in region (Nama Karoo and Little Karoo), which lay north of the GE and the Little Karoo area.

**Table 1:** The BioGeoBEARS results showing significant dispersal and vicariance events

Node	Dispersal	Vicariance	RASP route	Probability
	*Geographical regions*			
n4	Yes	Yes	BEF– > BFAEG– > BF|AEG	0.0144
n5	Yes	Yes	BF– > FBC– > F|BC	0.3614
n6	No	Yes	BC– > C|B	0.9835
n7	Yes	Yes	AEG– > EADG– > E|ADG	0.1716
n8	No	Yes	ADG– > G|AD	0.5493
n9	No	Yes	AD– > A|D	0.9960
	*Biomes*			
n4	No	Yes	AB– > A|B	0.6522
n5	Yes	No	A– > A^A– > AB^A– > A|AB	0.8410
n6	No	Yes	AB– > B|A	0.9062
n7	No	No	B– > B^B– > B|B	0.8281
n8	Yes	No	B– > B^B– > BC^B– > B|BC	0.7450
n9	Yes	Yes	BC– > ABC– > AB|C	0.8525
	*Geographic barriers*			
n4	Yes	Yes	A– > AC– > A|C	0.0932
n5	Yes	Yes	A– > AB– > A|B	0.3993
n6	Yes	Yes	B– > BA– > B|A	0.7221
n7	Yes	Yes	C– > CB– > C|B	0.3018
n8	Yes	Yes	B– > CB– > C|B	0.2287
n9	Yes	Yes	B– > BA– > B|A	0.5436

The hypothetical RASP habitat reconstruction history together with its probability is shown. The reconstruction used the seven geographic regions, three biomes and the three regions separated by the Great Escarpment and Swartberg Mountain. “ |” indicates geographic isolation, “ – > ” indicate directional changing of the RASP route

Our biome-based reconstruction analysis suggested the Nama Karoo and Succulent Karoo as the ancestral biomes, in which cladogenic events of *P. tentorius* took place before those in the Fynbos (see Fig. 6 and Table 1). Our BioGeoBEARs analysis suggested that in the case of node n4, the separation between the Nama Karoo and Succulent Karoo represented a significant vicariance event which influenced the cladogenesis of the *P. tentorius* species complex. It seems as though the northern population was derived from the Nama Karoo biome. The shifting of the latter biome in turn represented a vicariance event that split Ptr from Pv-B and led to the isolation of the Succulent biome. In the case of the southern population, it possibly originated in the Succulent Karoo, and the shift from Succulent Karoo to Fynbos may have been the vicariance event that separated Ptt-C from Ptt-B.

Our geographic barrier based analysis generally corroborated the findings of the geographic region based reconstruction, which suggests that the divergences at all nodes was strongly influenced by geographic barriers, by way of vicariance as well as dispersal (see Fig. 6 and Table 1).

### Diversification rate analysis

From the macro-evolutionary cohort matrices (Additional file 1: Figure S4), five comparatively distinct rate regimes have occurred in the *P. tentorius* species complex, (1) between Ptt-B and Pv-A, (2) between Ptt-B and Pv-B, (3) between Ptt-B and Ptr, Ptt-A and Ptt-D, (4) between Pv-B and Ptt-C, Ptt-D, Ptt-A and Ptr, (5) between Pv-B and Pv-A. The highest rate variation was found between Pv-B and Ptt-B + Ptr + Ptt-C + Ptt-A + Pv-A. The diversification rate between Pv-B and Ptt-D was lower than that of the other clades.

**Fig. 7:**
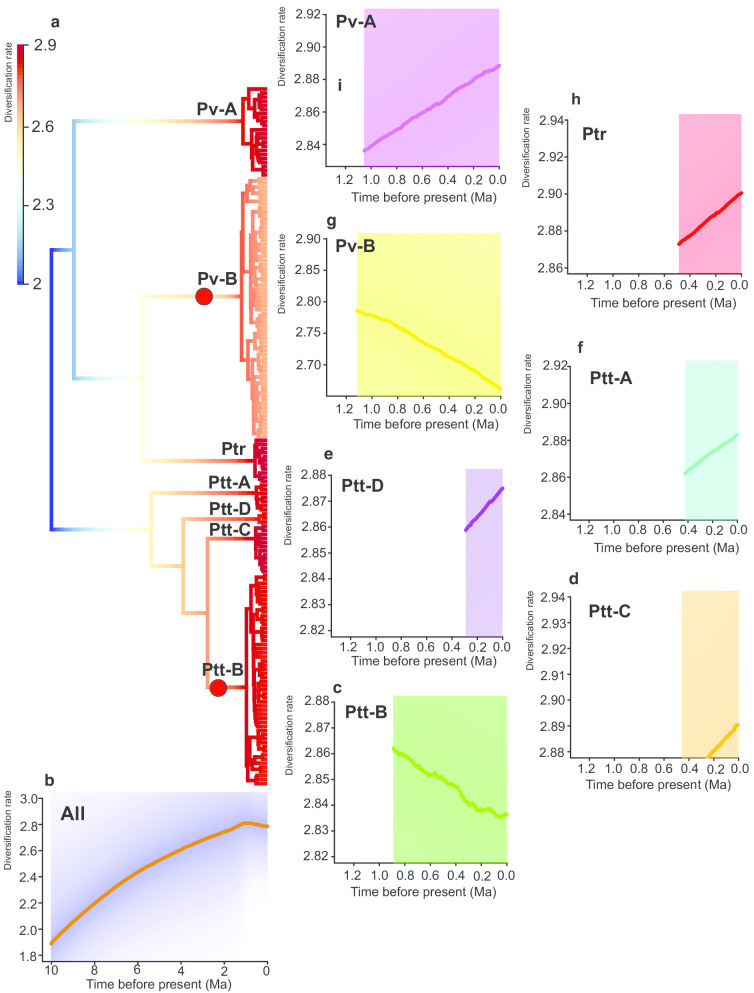
**a** The phylorate plot shows the haplotype diversification rate across the BEAST chronogram topology. The “warm” colours represent fast rates, whilst “cool” colours represent the branches with slow rates (units per event or lineages per Ma). The red spots indicate corresponding nodes with significant rate shifts. **b** The rate though time (RTT) plot for the overall *Psammobates tentorius* complex. **c**–**i** The RTT plots for different clades. The red dot indicates a significant rate shift detected by BAMM

The phylorate plot showed two clear evolutionary rate shifts, one at Ptt-B and the other at Pv-B (Fig. 7a). The Bayes Factors (BF) was highest at these two rate shifts scenario, being BF = 13.2. The rate through time (RTT) plot for the entire *P. tentorius* species complex showed a trend of increasing diversification rates after its cladogenesis started approximately 10 Ma, levelling off only in recent time (approximately 1 Ma, Fig. 7b). The RTT plots at clade level (Fig. 7c–i) revealed a trend of increasing diversification rates at Ptr, Ptt-C, Ptt-A and Pv-A, but especially at Pv-A (Fig. 7i). A clear decreasing trend was found at Ptt-B and Pv-B.

The BAMM results (Table 2) revealed Ptt-B and Pv-B as having substantially higher net diversification rates (diversification rate–extinction rate) and lower turn-over rates than the rest of the clades, though Pv-B exhibits the lowest diversification rate. All clades and groups had higher speciation rates than extinction rates. The overall lineage turn-over rate was relatively high in clades Ptr, Ptt-A–Ptt-D– and the two major branches “(Ptt-B + Ptt-C) + Ptt-D) + Ptt-A” and “(Pv-B + Ptr) + Pv-A”. The entire complex generally had a low overall net diversification rate and a relatively high lineage turn-over rate (~ 10 Ma to present). The plots of λ retrieved from the BAMM analysis results (Fig. 7) revealed a clear increasing diversification trend at Ptr, Ptt-C, Ptt-A, and Ptt-D, but especially at Pv-A (increasing λ in all cases), since the slope of linear relationship between λ and the time line in each case was significantly steeper than that in the other clades. By contrast, Ptt-B and Pv-B showed a remarkable decline in their λ plots, indicating a decreasing trend in net diversification.

**Table 2:** The estimated speciation rate (λ), extinction rate (μ), net diversification rate (r) and lineage turn–over rate (t) generated from the MuSSE analysis (for modelling the effect of geographic regions and biomes), BiSSE analysis (for modelling the effect of the Orange River) and BAMM analysis (for computing the overall diversification pattern for each target group)

		Diversification rate	Extinction rate	† Net diversification rate	‡ Lineage turn-over rate
Clade (BAMM)	Ptt-B	2.874	1.409	1.465	0.490
	Pv-B	2.745	0.830	1.915	0.302
	Ptr	2.908	2.439	0.469	0.839
	Ptt-C	2.910	2.315	0.596	0.795
	Ptt-A	2.874	2.444	0.429	0.851
	Pv-A	2.875	2.374	0.500	0.826
	Ptt-D	2.850	2.440	0.409	0.856
	(Ptt-B + Ptt-C) + Ptt-D) + Ptt-A	2.712	2.278	0.433	0.840
	(Pv-B + Ptr) + Pv-A	2.525	2.264	0.261	0.897
Overall	Entire complex	2.488	2.312	0.176	0.929
Region	1. Above GE	2.097	0.164	1.933	0.078
(MuSSE)	2. Below GE, north of Swartberg	2.011	1.037	0.974	0.516
	3. South of Swartberg	0.988	0.967	0.021	0.979
Biome	1. Nama Karoo	2.041	0.2	1.841	0.098
(MuSSE)	2. Fynbos	6.465	6.674	-0.209	1.032
	3. Succulent Karoo	0.852	0.536	0.316	0.629
Orange River	1. North of Orange River	0.779	0.703	0.076	0.902
(BiSSE)	2. South of Orange River	1.323	0.133	1.19	0.1

The unit used in the table was uniform for the cladogenesis events (or lineage events) per Ma

†Net diversification rate (r) = Speciation rate (λ)—Extinction rate (μ)

‡Lineage turn-over rate = μ / λ

**Fig. 8:**
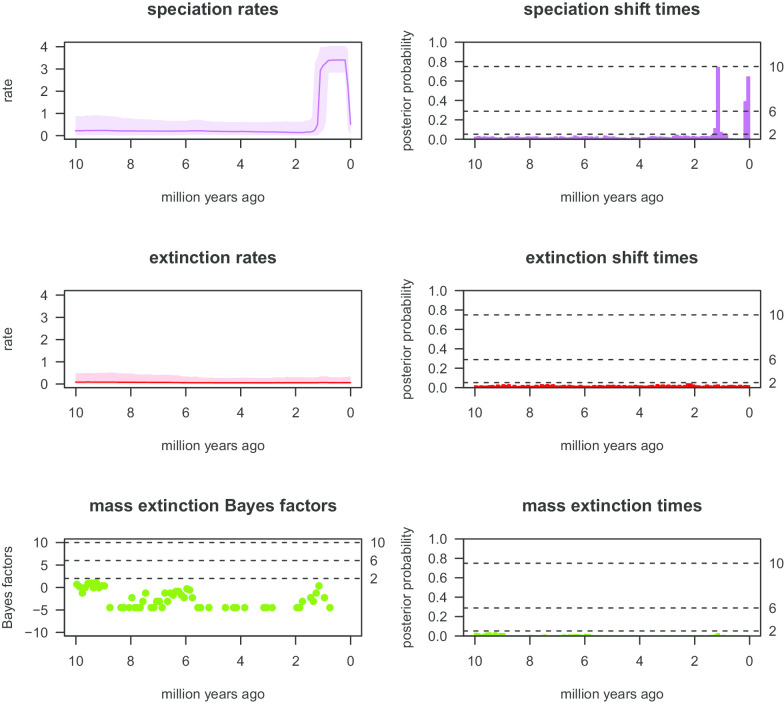
Visualization results of the CoMET analysis with empirically estimated diversification hyperpriors of speciation rates, extinction rates, Bayes factors of mass extinction events, the frequency of estimated mass extinction events, and the rate shift patterns of speciation and extinction

Our TESS analysis (see Fig. 8) results revealed clear increasing speciation rates during the Pleistocene (~ 2 Ma to present), with the speciation rates tending to decline thereafter, while two diagnosable speciation rate shift events were detected during the Pleistocene (~ 2 Ma to present), each exhibiting high frequency. No significant signal of extinction events, changing extinction rates or extinction rate shifts were detected. The Bayes factor plots also did not reveal any signal of mass extinction. Our single–chain MCMC diagnostic analysis confirmed the reliability of the CoMET analysis results and that sampling was adequate (see Additional file 1: Figure S5), since all the ESS values were larger than 200 and the Geweke statistic plot tended towards satisfactory.

### Dependent character analysis

The MuSSE analysis suggested that the GE and SM geographic barriers may have substantially influenced the historical diversification of the *P. tentorius* species complex, and identified the “full model” (λ, μ and q, which all differed across the three regions), with strong statistical support, as the best scenario (model likelihood scores and criteria provided as Additional file 1: Table S5). The biome based MuSSE analysis statistically confirmed that biomes affected diversification significantly, since the “full model” was favoured according to the LRT test. By contrast, results of the BiSSE analysis indicated that the OR did not influence the divergences in *P. tentorius*, because results for the best model, “free λ”, were not statistically significant.

**Fig. 9:**
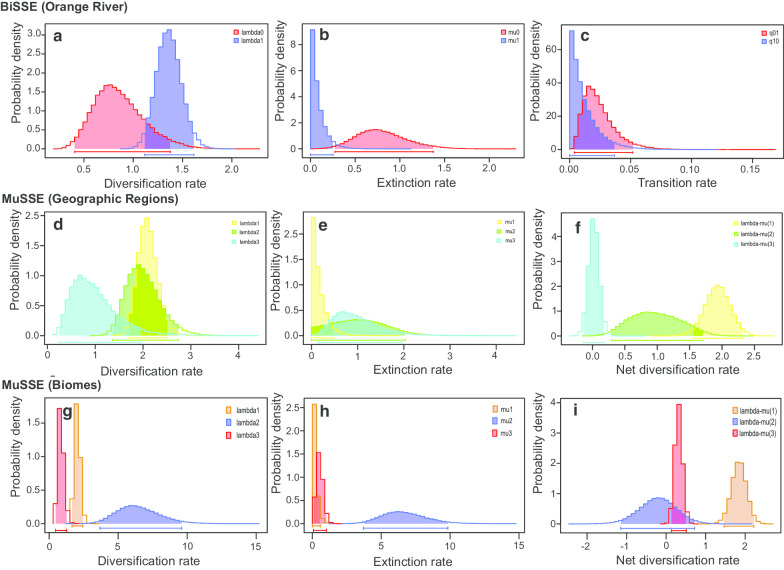
The estimated λ, μ and q (**a**–**c**) from the BiSSE analysis: “0”—populations from north of the Orange River, “1”—populations from south of the Orange River; **d**–**f**, MuSSE analysis results of three geographic regions: “1”—region above the GE, “2”—region south of the GE, and north of the SM and “3”—region south of the SM. Plots show λ, μ and net diversification rates (r) across the three different regions; **g**–**i**, MuSSE analysis modelling the λ, μ and r of three biomes: “1”—Nama Karoo, “2”—Fynbos mixed with Succulent Karoo, “3”—Succulent Karoo. Units were “events/Myr” in all cases

The BiSSE analysis results revealed a much lower net diversification rate, and higher lineage turn-over rate, in populations north of the OR than in ones south of it (Fig. 9 and Table 2). MuSSE analysis of regions indicated low net diversification rates and high lineage turn-over rates in populations south of the SM, and in populations occupying a mixture of Fynbos and Succulent Karoo vegetation (Fig. 9, Table 2).

### Environmental niche modelling

Our ENM analyses generally provided meaningful predictions of suitable ranges for the different groups of the *P. tentorius* species complex [in all cases, the average test AUC (under ROC) > 0.90 with a standard deviation < 0.06; for details see Additional file 1: Table S6], suggesting that the modelling process was reliable. The optimized parameter settings for each of the ENM analyses are given in Additional file 1: Table S7. Nevertheless, the mean omission rates of Ptt-A and Ptt-D showed relatively large deviations from the predicted omission rates (plot not shown), suggesting that the ENM range predictions for Ptt-A and Ptt-D should be interpreted with caution. Possible reasons for this may be their smaller ranges and smaller sample sizes.

**Fig. 10:**
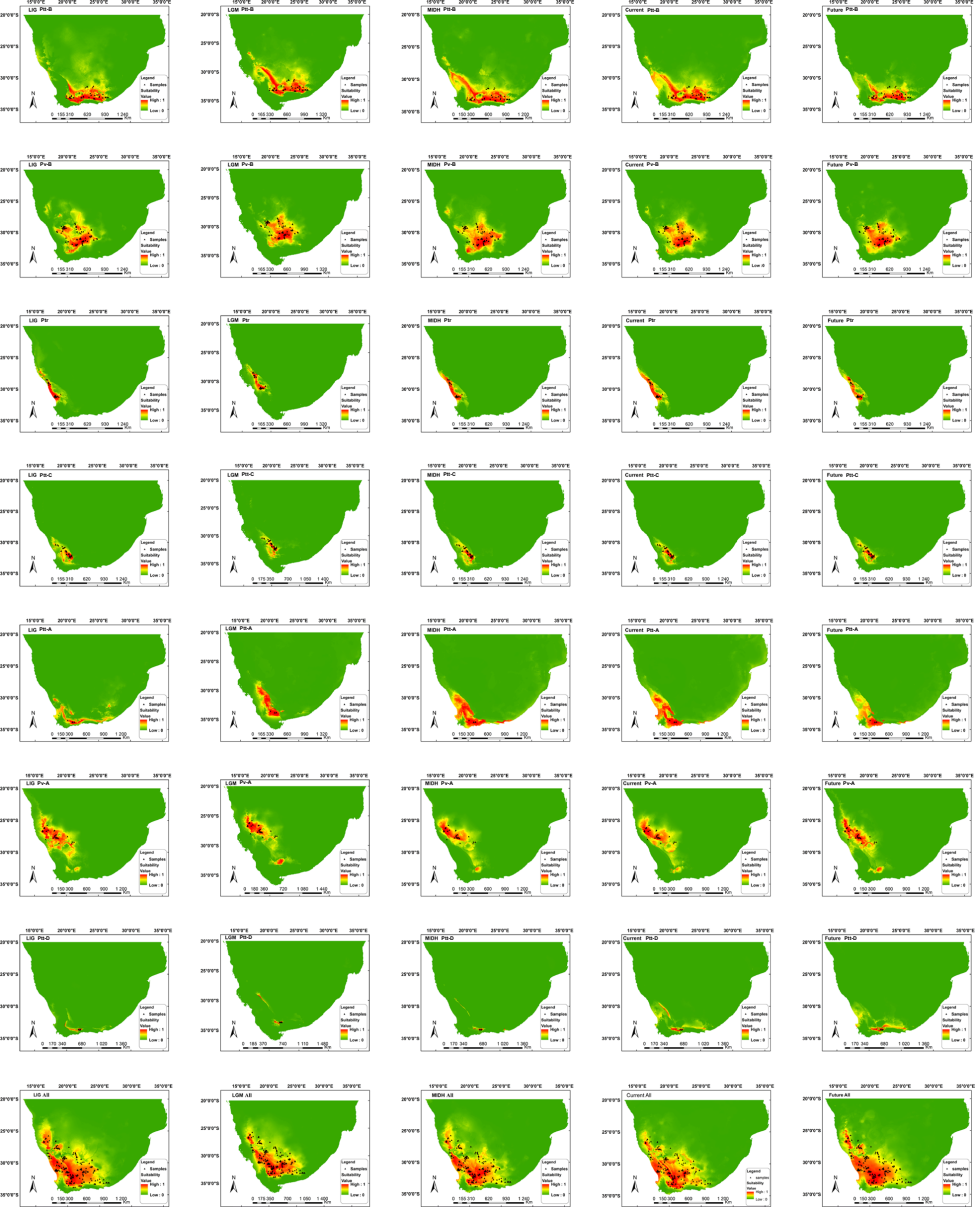
The potential ranges of suitable habitat for each of the seven clades of the *P. tentorius* species complex, combined in Southern Africa across different periods ranging from the LIG to the future (up to 2070). Environmental niche modelling was done with present bioclimatic variables on the basis of extant points of occurrence (black triangles) of the species, using the Maxent program. Information about clades and periods are given at the top–left corner of each analysis

The majority of the groups received relatively meaningful range predictions, which aligned well with their currently recognized distribution ranges (see Figs. 1 and 10), but not with the niche boundaries between Ptt-B and Ptt-C, Ptt-C and Ptt-A, Ptt-B and Ptt-D, and Ptt-A and Ptt-D, which are still fuzzy. The pairwise niche significance analysis results based on I statistical criterion found no significant differences between Ptt-B and Ptt-A, and between Ptt-B and Ptt-D, while all the other pairwise comparisons between clades were significant (see Additional file 1: Table S8).

Overall, when comparing the ENM analysis results across timelines, many groups exhibited substantial shrinking of suitable habitats in the Last Glacial Maximum (LGM) period (see Fig. 10 and Additional file 1: Table S9) compared to the Last Interglacial (LIG) period. When comparing the size of currently suitable habitats to that in the Middle Holocene (MIDH), an overall declining trend was observed for the *P. tentorius* species complex, however, the decline was only significant in case of Pv-B, Ptt-C and Ptt-A (Additional file 1: Table S9). As far as predicting future range shifting scenarios is concerned, Ptt-B, Pv-B, Ptr, Ptt-A and Pv-A revealed substantial declines in suitable habitats, with only Ptt-C and Ptt-D exhibiting increasing trends in suitable areas. It is noteworthy that Pv-B and Ptt-A showed continuous declines in suitable areas since the MIDH period.

**Fig. 11:**
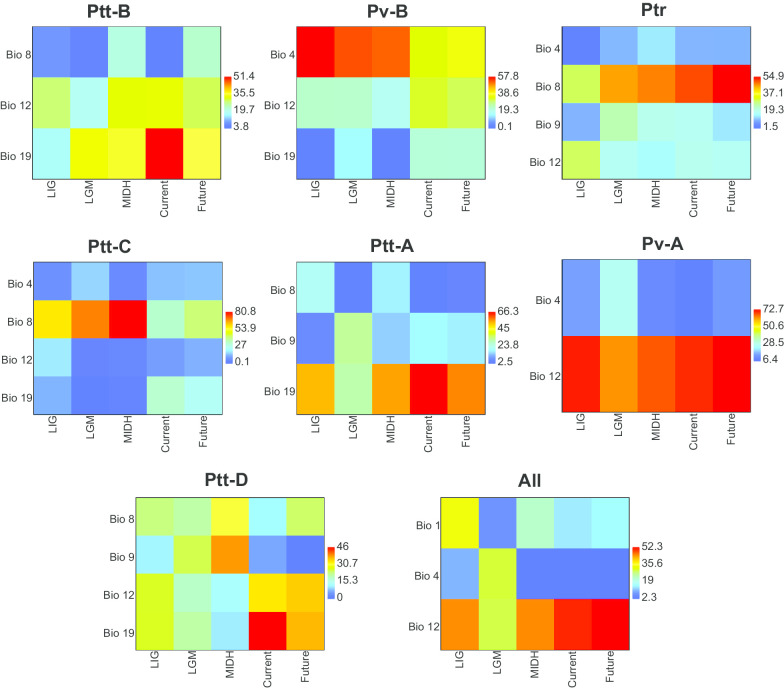
Heatmaps visualizing the changing impact contributed by each critical climatic variable (Jack-knife AUC > 0.75) against the timeline (ranging from the LIG to the future) across all groups (on each of the seven clades independently and combined). The warm colours represent climatic variables with high influence on niche modelling, whilst, cold colours represent low impact; the numbers in the legend are percentages

In terms of climatic variables that made an impact in the ENM analyses (for details, see Additional file 4: Table S10 and Fig. 11), substantial differences were found among clades and across different periods. In Ptt-B, Ptt-A and Ptt-D, precipitation of the coldest quarter had the greatest influence on their habitat suitability. Temperature seasonality was the most influential climatic variable of the habitat suitability pattern in Pv-B. For Ptr and Ptt-C, mean temperature in the wettest quarter contributed the most to their habitat suitability pattern. The annual precipitation was the most important climatic variable shaping habitat suitability patterns in the *P. tentorius* species complex collectively. However, when the clades were assessed independently, it was only applicable to Ptt-B–Ptt-C, Pv-A and Ptt-D. In most cases, the LGM period appeared to be more greatly impacted by the changing climatic variables. Overall, the annual precipitation, mean temperature of the wettest quarter, temperature seasonality, and the precipitation of the coldest quarter were the four most important factors that shaped the habitat suitability patterns for the tent tortoises.

## Discussion

### Genetic structure and phylogeny

Overall, our microsatellite DNA based DAPC clustering analysis results corroborated the sequence DNA (mtDNA + nDNA) based phylogeny, giving a similar genetic structure pattern of the seven clades. However, our STRUCTURE clustering analysis did not reveal diagnosable differences between Ptt-B and Ptt-C, and between Ptt-A and Ptt-D (Fig. 3b–c). According to Zhao et al.[40], some of the species delimitation analyses also revealed that the species boundary between Ptt-B and Ptt-C was not clear, thus treating Ptt-B and Ptt-C as the same candidate species is certainly reasonable. However, treating Ptt-A and Ptt-D as a single candidate species or OTU, will conflict with our phylogenetic structure, since the DNA sequence based phylogeny did not support Ptt-A and Ptt-D as a sister group. Furthermore, the assigning of OTU or species status should not purely rely on genetic markers, but on multiple lines of evidence which includes comparative morphology and ecology [44, 53]. Our Migrate analysis generally revealed a limited amount of gene flow among the four clades (Ptt-B, Ptt-C, Ptt-A and Ptt-D) in the southern group, which may imply substantial genetic isolation among these clades. All of these clades of the southern group were isolated by geographical barriers (mountains), which may have facilitated genetic divergence. The results indicated that the genetic differences among these clades were substantial. However, the estimated gene flow level was comparatively high between Ptt-B and Ptt-C, suggesting that the degree of genetic isolation between them was low. Nonetheless, the genetic difference between the two clades was found to be significant, based on multiple species delimitation analyses in Zhao et al. [40], and was attributed to the western section of the GE completely separating them geographically. This evidence supports the genetic distinctiveness of Ptt-B and Ptt-C. In the northern group (Pv-B, Ptr and Pv-A), gene flow levels among the three clades were also comparatively low, indicating substantial genetic divergence among them. Each clade occurs in a different biome [54], the western section of the GE separates Pv-B and Ptr geographically, and the Orange River is a major geographic barrier between Pv-A and (Pv-B + Ptr).

Regarding the intergradation zones between Ptt-B and Pv-B in the “biological corridor” of the Upper Karoo, and between Pv-B and Ptt-C in the western Nama Karoo, our Migrate results revealed substantial amounts of geneflow between Ptt-B and Pv-B, and Pv-B and Ptt-C. The directional gene flow rate estimated from Migrate revealed that the magnitude of gene flow from Ptt-B to Pv-B was substantially greater than the rate from Pv-B to Ptt-B, whilst, the magnitude of gene flow from Pv-B to Ptt-C was also substantially greater than the flow rate from Ptt-C to Pv-B. These results obtained from the directional gene flow rates corroborate our present phylogeographic findings (in terms of distribution of the clades) that Ptt-B is present above the GE, but that Pv-B is not present below the GE. Ptt-C is only present in the Succulent Karoo and Fynbos, not the Nama Karoo, but Pv-B can be marginally present in Succulent Karoo. The Migrate results also highlighted possible hybridization between Ptt-B and Pv-B, and Pv-B and Ptt-C, but it requires further verification with extensive sampling in these overlapping zones. Furthermore, if hybridization is possible, it will have to be determined whether the potentially hybridized individuals are also able to produce viable offspring.

Our ENM analyses, for example, showed that the niche overlaps among clades of the southern group (Ptt-B, Ptt-C, Ptt-A and Ptt-D) was substantial, and that their ecological niches did not differ significantly. Therefore, when deciding on the most parsimonious strategy, based on genetics and ENM, for assigning OTU status that would be crucial to its conservation management, we recommend that four OTUs or candidate species be recognized in the taxonomic revision of the *P. tentorius* species complex, namely, Pv-B, Ptr, Pv-A and (Ptt-B + Ptt-C + Ptt-A + Ptt-D). Furthermore, as a morphological character based study is in progress (Z. Zhao et al. in prep), we suggest that the final taxonomic decisions also take into account those findings.

### Diversification patterns and biogeography

Cladogenesis in *P. tentorius* started during the early to late Miocene, a period initially characterised by warm temperatures [6], which may have facilitated dispersal of ancestral *P. tentorius* over a relatively wide area of southern Namibia and northern South Africa. Ancestral area analyses showed that region BEF (Nama Karoo and Little Karoo) was the most likely regions of origin. The absence of fossil records in the biometric analysis may, however, have compromised the accuracy of estimates, consequently, the divergence dates should be treated as ranges rather than means.

Our calibration dating analyses suggested that the first divergence that split *Stigmochelys pardalis* from genus *Psammobates* matched a global cooling event at the Eocene–Oligocene boundary (approximately 33.9 Ma), which is believed to have led to aridification and major extinctions in many organisms, but without causing changes in biome composition [55]. This event may have led to range contractions, which facilitated the divergence between *Psammobates* and *Stigmochelys*. It also suggests that the split between *Psammobates* and *Stigmochelys* may not have been caused by biome shifts, but rather by climate change, although there might be a correlation or interactions between biome shift and climate change, the climate change inducing the biome shift [4, 8].The timespan when the *P. tentorius* ancestor diverged from other *Psammobates* species presumably covered the early Miocene, when temperatures dropped and the Antarctic ice-sheet formed [6]. In addition, the opening of the Drake Passage caused cold water (Benguela Current) to flow northwards along western South Africa [56] so that the southern part of Africa became progressively more arid [7]. This cooling and gradual aridification possibly contributed to diversification and the separation of ancestral species into distinct geographical regions. Based on our combined total evidence dating results, *P. tentorius* filled the geographic space between *P. geometricus* in the southwest and *P. oculifer* in the north, suggesting that the ancestor of the three species possibly had a wide distribution across Southern Africa during the early Miocene, and that global cooling in the early Oligocene likely restricted their ranges and isolated populations. Evidence shows a significant global temperature decline during the 25–23 Ma interval [6, 57]. This cooling event possibly led to the contracting of ranges and refugia, resulting in the separation between *P. tentorius* and other *Psammobates* species. The divergence between *P. geometricus* and *P. oculifer* dates back to the early Miocene, coinciding with a warm and humid period (23–16 Ma, [57, 58]). The warm and humid climate may have resulted in rapid changes in vegetation and habitats, which may have driven the divergence between *P. geometricus* and *P. oculifer*. Since the time of formation of the biomes perfectly matched the beginning of the cladogenic diversification in *P. tentorius* [4, 59, 60], it is possible to speculate about the geographic region in which each taxon developed.

Divergence at node n4 divided the ancestral species into two lineages, a northernmost clade (Pv-B + Ptr + Pv-A) and a southernmost clade (Ptt-B + Ptt-C + Ptt-A + Ptt-D). The “DIVALIKE + J” RASP route analysis indicated that the range of the ancestral group first became restricted to the upper Nama Karoo and western Little Karoo, from where a southwards dispersal occurred to the lower Nama Karoo and eastern Little Karoo. The final step was a divergence between populations of the upper Nama Karoo and those south of the GE, indicating a geographic split between the northern and southern lineages. From this scenario it would appear that ancestral *P. tentorius* crossed the OR despite it representing a potential geographic barrier. The analysis suggested that both vicariance and dispersal played a role in this first divergence. The biome-based reconstruction did not show significant geographic conflicts, and also revealed a significant vicariance event caused by biome segregation between the Nama Karoo and Succulent Karoo, suggesting that biome shifting also drove the first divergence. Soon after the *P. tentorius* ancestor divided into two lineages, Pv-A diverged from Pv-B + Ptr at node n5. The DIVALIKE + J RASP route analysis indicated a second crossing of the OR from the north, followed by dispersal into the west coast and the upper Nama Karoo on the southern side of the OR in South Africa, and finally a divergence between populations north and south of the OR. This divergence apparently involved both dispersal and vicariance. The biome changes does not seem to have influenced cladogenesis by vicariance at this point, but rather facilitated dispersal.

Within the *P. tentorius* species complex, diversification was possibly initiated in the late Miocene (approximately 9.98 Ma), so it seems as though the mid Miocene Climatic Optimum [58] was not responsible for the diversification of *P. tentorius*. Instead, the cladogenic events of *P. tentorius* seem to have coincided with the late Miocene cooling, intensifying of aridification of Southern Africa [61], the late Miocene global diversification of succulent plants [61] and also the Miocene–Pliocene stepwise intensification of the Benguela upwelling [62]. The late Miocene cooling and intensifying of aridification may thus have caused habitat constriction and the retracting of refugia, which possibly initiated the diversification of the *P. tentorius* species complex. As succulent plants are the major food source of *P. tentorius* [22, 63], their diversification may have influenced its habitat range substantially. The fragmentation of habitats with suitable succulent plants may in turn have resulted in isolation of subpopulations [61], providing the essential driving force for diversification. Furthermore, the intensification of the Benguela upwelling (at approximately 10 Ma) may have resulted in increasing aridity on the western side of South Africa [62], where the majority of populations of *P. tentorius* occurred, particularly along the western costal area. As discussed above, the increasing aridity may have resulted in the retracting of ranges and refugia, which possibly led to its speciation. Several studies using different methodologies indicated that major uplift events in Southern Africa occurred during the Cretaceous but that tectonic uplift during the Cenozoic was at a relatively small scale [64–66]. Yet, uplifts of up to 250 m during the Miocene [67, 68] may have influenced the distribution routes of *P. tentorius*. The character dependency analyses suggested that the OR did not play a significant role in cladogenesis events, and that vicariance resulted from climate changes rather than from the development of the OR. Equally for node n5, we propose that dispersal related to *P. tentorius* crossing the OR for a second time and dispersing into the northern regions of South Africa, while vicariance resulted from populations retracting into refugial areas in response to a steep reduction in temperature after the late Miocene cooling [6]. The western GE in Namibia and South Africa may have served as both refugia and corridors for diversification in *P. tentorius*, as it had for other taxa [69]. More research is necessary to see if members of the clades still cross the OR on occasion and perhaps hybridize.

There are few reptile taxa with similar distribution patterns as *P. tentorius* in Southern Africa, for which divergence dates have been estimated. Nevertheless, Tolley et al*.* [70] proposed that the deep divergences among major clades of the genus *Bradypodion* date back to the late Miocene, and implicated vicariance associated with both geological and climatic events. The latter included uplift of the western GE [68], formation of the Benguela upwelling system and late Miocene cooling [71]. Furthermore, major cladogenic events in the skink genus *Trachylepis* were also found between the mid- to late Miocene cooling [72].

The next two divergences, at nodes n6 and n7, occurred more or less at the same time in the late Miocene, with both divergences being ascribed to vicariance, and in the case of node n7 also to dispersal. The divergence at node n6 involved the separation of Ptr on the western coastal plains from Pv-B above the GE. The most likely explanation for this cladogenic event is increasing western aridity due to the intensification of the Benguela upwelling in the period from 6.2 to 5.5 Ma [9, 62, 73], which perfectly matches the divergence time between Pv-B and Ptr. In parallel with western aridification, the initiation of a winter rainfall regime and the development of Succulent Karoo vegetation on the west during the late Miocene, came to full development in the Pliocene [7, 8, 21]. Our biome-based reconstruction results also corroborate this finding, of a vicariance event caused by the separation of the Nama Karoo and Succulent Karoo biomes which may also have facilitated the divergence between Pv-B and Ptr. Vicariance due to climate thus seems more likely than the western GE developing into a physical barrier between the coastal plains and interior plateau. The GE undoubtedly would have restricted gene exchange, but there are low-lying regions, particularly in the northwest, which could have served as dispersal routes between the coastal plains and plateau, and thus between Ptr and Pv-B. Nevertheless, there is evidence for a minor uplift in the west in the early Pliocene [68], which possibly strengthened isolation between the two clades.

*Psammobates tentorius* dispersed to the southern region of South Africa by the early Pliocene. The first divergence at node n7 isolated Ptt-A in the western Little Karoo from the rest of *P. t. tentorius*. Thereafter, Ptt-D in the eastern Little Karoo Oudtshoorn basin diverged from *P. t. tentorius* clades 1 and 4, in the lower Nama Karoo, and the eastern side of the Hantamberge and Roggeveldberge above the GE. In both instances, vicariance seemed to have been of primary importance, although dispersal is also suggested. Both geographic region and geographic barrier based reconstruction analyses supported these assumptions. These divergences may have been caused by either climate change or Cenozoic exhumation [14, 74]. In particular, uplift intensified greatly in the last 5 Ma for the GE alone, its highest elevation point rising from 300 to 900 m. Therefore, the intensification of uplift may have facilitated geographical isolation. Since climatic and vegetational changes were not restricted to the Little Karoo, it seems unlikely that these changes could have played a major role in vicariance. Our biome reconstruction analysis also implied that biome shifting did not contribute significantly to cladogenesis at node n7 and node n8, despite a minor contribution from dispersal at node n8.

Evidence for the effects of geomorphological changes on vicariance in the southern clades seemed strong. Thermochronology results indicated that regions west and east of Worcester in the southwestern Cape underwent burial (up to 1.2 km) prior to exhumation (starting 30–20 Ma), which created the current relief with surrounding peaks reaching elevations of 1500 m [14]. It seems likely that over time this exhumation isolated *P. t. tentorius* populations in the Little Karoo from their western and northern counterparts. In addition, thermochronology results indicated differential uplift followed by exhumation (starting 30–20 Ma) in mountain ranges of the Oudtshoorn basin [14]. These events could have isolated Ptt-D of the eastern Little Karoo from Ptt-A of the western Little Karoo. Despite being on a more recent time scale, divergence within a Little Karoo endemic plant species, *Berkheya cuneata*, shows two distinct lineages associated with the western and eastern Little Karoo, respectively [75], similar to Ptt-A and Ptt-D of *P. t. tentorius*. Pygmy geckos (*Goggia*) also show a similar differentiation pattern in the southern Cape [26]. On a broader scale, Cowling et al*.* [20] proposed that increased topographic heterogeneity in response to moderate uplift in the Miocene played a major role, together with climatic deterioration, in the rapid diversification of the Cape plant clades.

The final divergence between Ptt-C and Ptt-B, respectively, on the populations of western GE and below the southern GE, occurred during the late Pliocene with vicariance as the main driver, possibly due to both tectonic and climatic events. The late Miocene and early Pliocene (7.7–4.0 Ma) was relatively humid [76], which may have facilitated movements and gene exchange between populations below and above the southern escarpment. Also, as discussed above, the uplift intensified substantially in the last 5 Ma (for the GE alone), which may have facilitated geographic isolation. A possible contact zone may have been the relatively low slopes of the GE in the northern Tankwa Karoo, southwest of Calvinia. The Tankwa Karoo is currently one of the most arid parts of the Karoo with < 100 mm rain per annum [77]. Aridification may have closed this contact zone and isolated Ptt-B and Ptt-C from each other. Green et al. [14] provide evidence for a Cenozoic uplift of around 700 m and subsequent denudation in the Beaufort West GE region, but there is no evidence suggesting that this tectonic event was localised or affected the remaining southern escarpment. Nevertheless, if such an uplift influenced dispersal routes across the GE, it should have contributed to this diversification event. Noteworthy about this is that our ENM results also predicted a substantial contact zone in the Tankwa Karoo between Ptt-B and Ptt-C, which supports our assumption. Our Migrate results also revealed a substantial level of gene flow between Ptt-B and Ptt-C, which implies that the divergence between the two lineages was a fairly recent event. More importantly, this Tankwa Karoo contact zone between Ptt-B and Ptt-C appeared to show a substantial declining trend from LIG to the recent and future, which may imply that the further diversification between these two clades will be reinforced.

The phylogeographic analyses detected several Pleistocene diversification within Ptt-B, Pv-B, Ptt-C and Pv-A, while the lack of fine structure in Ptr, Ptt-A and Ptt-D may be due to small sample sizes. Periodic climatic cycles in response to Milankovitch forces caused glaciers to wax and wane over the Pliocene and Pleistocene [78], which influenced species distribution and diversification worldwide [2]. Divergences within Pv-B and Pv-A were possibly driven by the southward expansion of Kalahari sands in response to Pleistocene glaciation [16], as has been shown for the Namaqua rock rat (*Micaelamys namaquensis*) [79] and Chacma baboons (*Papio ursinus*) sensu lato [80].

Generally, our reconstruction analyses revealed that each clade occurred in a unique geographic region, suggesting that both biome shifting and geographic barriers played important roles in the cladogenesis of *P. tentorius*. Furthermore, the biome-based reconstruction analyses imply that biome formation and distribution may not be a once-off event, and that biome shifting did not necessarily correlate with allopatric speciation in *P. tentorius*.

### Diversification rates, potential cladogenic drivers and regional patterns

The diversification in *P. tentorius* was apparently not influenced by the OR but differed among regions and among biomes. The latter results, however, become more complex when considering the association between clades and regions or biomes. The lineage turnover rate was low for the region above the GE, which is paralleled by a low turnover rate for Pv-B, but not for Ptt-C or Pv-A. Lineage turnover rate was also low for the Nama Karoo, as well as for Ptt-B and Pv-B associated with this biome, but the turnover rate for Pv-A in the Nama Karoo north of the OR was high. The region and biome analyses were not very informative possibly because the division of characters oversimplified the great climatic and topographic heterogeneity of the Southern African landscape [40, 81].

Net diversification rate for the entire *P. tentorius* species complex was low but lineage turnover rate was high. Low net diversification rates and high lineage turnover rates applied to all clades, except Ptt-B and Pv-B, occurring in the Nama Karoo in central South Africa. The high net diversification rates of Ptt-B and Pv-B suggested that these clades had accumulated much diversity over a relatively short period of time, possibly due to a lower extinction rate rather than a higher diversification rate than in the other clades. Yet, the evolutionary dynamics of Ptt-B and Pv-B differed, with significant rate shifts in both clades associated with an increase in rate for Ptt-B and a decrease in rate for Pv-B. Species distribution models for Puff Adder (*Bitis arietans*) indicated that the interior of South Africa became inhospitable for this species during glaciation periods, and that populations continuously retracted and expanded to and from refugia in the northern and southern regions of South Africa [82]. We propose that Pv-B retracted northward during glaciation periods and that the relatively low landscape heterogeneity of their habitat (Bushmanland and Upper Karoo bioregions) reduced rate shifts in it. Our ENM results also supported the increasing range suitability from LIG to the recent in the northern population. In contrast, Ptt-B possibly retracted to southern regions near the CFMs in the south, which include several vegetation types such as the Lower Karoo bioregion, Albany Thicket and Fynbos. This also corroborates our ENM results (from LGM to recent). The higher topographic and vegetation heterogeneity of the southern region may explain the increase in rate shift in Ptt-B but a decreasing rate shift in Pv-B.

The evolutionary dynamics were similar for Ptr, Ptt-A, and Ptt-D below the GE, and for Ptt-C and Pv-A above the GE. The two biodiversity hotspots of the GCFR cover the full distribution ranges of four *P. tentorius* clades (Ptr, Ptt-C, Ptt-A and Ptt-D), and part of the ranges of Ptt-B and Pv-A (see Figs 1, 2 in [83]). Topographic diversity in the habitats of Ptt-C, Ptt-A, Pv-A, and Ptt-D is high but low for Ptr, except in the Richtersveld in the north, which includes the arid western escarpment [69]. Associated with this topographic diversity is high climatic diversity, with a strong aridity gradient from north (very arid) to south (more mesic) along the western, winter-rainfall region of South Africa [13]. The southern region is arid south and north of the SM, but becomes more mesic towards the east, and topographic heterogeneity creates localized climates [81] over the range of the southern clades. North of the OR, the habitat of Pv-A is also diverse not only with regard to topography but also climate. The southwest is highly arid and receives winter rains, whereas rainfall increases towards the east is received in summer rain [84]. The topographic and climatic heterogeneity of the abovementioned regions probably explain their similarities in evolutionary dynamics, characterised by high diversification rates, countered by high extinction rates, culminating in low net diversification and high lineage turnover rates.

Our TESS analysis results generally corroborated the findings of the BAMM analyses, and also revealed a substantially increasing speciation rate in the Pleistocene (~ 2 Ma to present), and a clear decreasing rate thereafter. The TESS analysis however revealed a more conservative estimation of the extinction rate. It did not reveal any diagnosable extinction event, changing extinction rate with time or shift in extinction rate across the cladogenic history of the *P. tentorius* species complex. These results therefore suggest that the extinction events and their rate changes estimated by the BAMM analysis could be an artefact. Interpretation of extinction events should therefore be done with caution, and verifying the true picture with palaeontological evidence (e.g. fossils and geology) is crucial.

The GCFR is recognised for its great floristic richness and endemism [42, 43], with much of its present diversity ascribed to cladogenic events during the Pliocene and Pleistocene [5]. The Pliocene–Pleistocene glacial cycles involved fluctuations between cool–arid conditions during glacial periods and warm–humid conditions during interglacial phases [1, 6], causing the contraction and expansion of taxa in and out of refugial areas. Several studies suggested the existence of western and eastern refugia in the southern GCFR [82, 85], as well as refugial areas in mountains of the western GCFR, such as the Cederberg and Kamiesberg [69, 86]. It appears that large patches of Fynbos and Succulent Karoo biomes persisted as refugia over glacial/inter–glacial cycles, which was not the case with the Nama Karoo; recent expansion of the Nama Karoo may thus explain present contact zones of many taxa [5] and references therein. Such contact zones for *P. tentorius* include intergradation zones between Ptt-B and Pv-B, and between Pv-B and Ptt-C, which in both instances may have involved recent expansions of Nama Karoo clades.

It is noteworthy that time-calibrated chronograms of extant species are widely used for investigating diversification patterns and dynamics [87]. However, the reliability of these inferences is still under debate [87–91]. A recent study by Louca and Pennell [91] suggested that purely time calibrated chronogram-based diversification rate analyses did not generate reliable results for inferring diversification dynamics in the absence of biologically well-justified constraints or additional palaeontological information. Many previous studies may therefore have misinterpreted the outcomes of such diversification rate analyses and the phylogenetic signal. The findings of Louca and Pennell [91] therefore highlighted the importance of using reliable palaeontological data for answering macroevolutionary questions. Accordingly, the interpretation of diversification dynamics of the *P. tentorius* species complex therefore requires supporting fossil evidence. In our study, the source priors of the constrained nodes for divergence time estimations came from phylogenetic studies that used fossil records. Our time–trees may therefore be considered as reliable, though further fossil-based evidence is crucial to further verify the inferences of diversification dynamics made in this study.

### Perspectives on potential impacts of climate and environmental change

Our ENM results revealed that clades Pv-B, Ptr and Pv-A occurred in unique niches. The niche boundaries between Ptt-B, Ptt-C, Ptt-A and Ptt-D are, however, not clear. These results imply that Pv-B, Ptr and Pv-A occur in unique habitats, influenced by different climatic and environmental factors compared to clades Ptt-B, Ptt-C, Ptt-A and Ptt-D. The latter four clades occur in habitats shaped by similar climatic and environmental factors. The Tankwa Karoo region (southwestern Karoo) was regarded as a “three-way” contact zone, where all three currently recognized subspecies were believed to occur, given the high polymorphism in morphology found in one area [92]. Zhao et al. [40], however, found that all these morphs belonged to the same clade, Ptt-B (supported by both mtDNA and nDNA sequence data). Our ENM analyses revealed substantial overlapping zones of suitable areas in the Tankwa Karoo for Ptt-B, Ptt-C and Ptt-A (corresponding to *P. t. tentorius*), Pv-B and Pv-A (corresponding to *P. t. verroxii*). This interesting finding implies that the high phenotypic variation in the Tankwa Karoo population was possibly driven by convergent evolution due to high microclimatic heterogeneity, making the Tankwa Karoo suitable for all these clades.

Clearly, the recent Pleistocene climatic oscillations (from the LIG to LGM period, but particularly in the LGM) functioned as important evolutionary drivers that impacted distribution pattern. Areas with suitable habitats and potential ranges for the *P. tentorius* species complex were primarily based on our ENM analyses, with range shifts becoming particularly pronounced in the LGM period. Although there was an overall decline in the potentially suitable range area from the LIG to LGM for the *P. tentorius* species complex overall, possibly due to cooling which led to the retracting of ranges and refugia, the decline was significant only in the case of Ptr, Ptt-C, Pv-A and Ptt-D. The reason for the increasing ranges of Ptt-B, Pv-B and Ptt-A is unclear, but may be correlated with variations in localized climates during the LGM. Studies in tropical and subtropical areas found that different habitat shifting scenarios could shape glacial refugia [1, 93]. Noteworthy about our ENM results was that they revealed a diagnosable subdivision within Pv-A (between western and eastern populations), which seems to have been reinforced since the LGM cooling. This was also observed in the microsatellite based cluster analysis and DNA sequence based phylogeny. Besides this, our haplotype based diversification rate analysis revealed an increasing trend of diversification that corroborated our ENM results for Pv-A. The predicted potential range exhibited an overall decreasing trend. This is probably related to environmental gradients that limited homogenizing through gene flow or it could be a refugial area that has persisted over time, resulting in a build-up of diversity. The range shrinking and refugia retracting can facilitate isolation and speciation [1, 94]. Collectively, it implies that further divergence within Pv-A is very likely. This was also advocated by [40], who found that genetic diversity within Pv-A was comparatively high.

When comparing the results of diversification rate analyses (Fig. 7) and ENM analyses in the LGM (Additional file 1: Table S9), an interesting trend was observed, namely, that a decrease in suitable range generally correlated with an increase in diversification rates, and vice versa. This trend is in-line with the theory that cooling results in the retracting of ranges and refugia, which facilitate diversification and speciation.

Our ENM analyses revealed that range suitability of each clade was shaped by different combinations of climatic variables. The impact of climatic variables at different periods was also different for each clade. The LGM was the period in which the climatic variables had their most significant impact on most clades (see Fig. 11). It reflects the high level of ecological differences among clades, particularly between the northern (Pv-B, Ptr and Pv-A) and southern groups (Ptt-B, Ptt-C, Ptt-A and Ptt-D).

Overall, potentially suitable range for the *P. tentorius* species complex appears to be strongly influenced by annual precipitation, temperature and temperature seasonality, with annual precipitation as the most important one. Rainfall patterns determine primary productivity (plants), and is therefore strongly correlated with the distribution of vegetation and biomes. This is especially crucial for *P. tentorius*, as a herbivore [22]. These findings also corroborated those of our biogeographic analyses that cladogenesis was substantially influenced by biome and vegetation characteristics. Some climatic variables were, however, filtered out after the correlation analysis, which may also influence range suitability, making further ecological studies crucial to unmasking the full ecological picture.

### Prospective conservation management plan

Since *P. t. tentorius* (Ptt-B, Ptt-C, Ptt-A and Ptt-D) and *P. t. verroxi* (Pv-B and Pv-A) have been categorized as “Near Threatened”, and *P. t. trimeni* (Ptr) downgraded to “Endangered” by the IUCN in 2018 [95], they all deserve conservation attention. Our ENM analyses showed a substantial decline in the current suitable range of Pv-B, Ptt-C and Ptt-A, and also predicted a substantial declining trend for Ptt-B, Pv-B, Ptr, Ptt-A and Pv-A by 2070. Both Ptt-B and Pv-B also showed consistent decreases in diversification rates. These findings highlight the need for conservation attention at the very least for Ptt-B and Pv-B. Apart from the influence of varying climate, there are also anthropogenic factors such as urbanisation which will likely reinforce future range declines, particularly in the southern group, where such activity is high.

Although most of the range of Ptr falls in a protected area (Namaqua National Park), the findings support the prediction of Branch [22], that it will be impacted by ongoing climate change. The distribution ranges of Ptt-B, Pv-B and Ptt-C overlap with those of other species listed by the IUCN in 2018 [95], namely, the Karoo dwarf tortoise (*Chersobius boulengeri*, “Endangered”), speckled dwarf tortoise (*Chersobius signatus*, “Endangered”) and the armadillo girdled lizard (*Ouroborus cataphractus*, “Vulnerable”), so they could therefore indirectly benefit from conservation attention given to those taxa. An area, for example, warranting preservation in this regard is the biological corridor between the Nuweveldberge and Sneeuberge, where Ptt-B and Pv-B coexist along with the sympatrically occurring Karoo dwarf tortoise. Unfortunately this area is not protected yet.

The home range of Pv-A certainly deserves conservation attention, given that it has the highest genetic potential for further cladogenesis. Its distribution range overlaps with that of the Nama dwarf tortoise (*Chersobius solos*) categorized as “Vulnerable” [95]. Fortunately, the region already has protected areas like the Ai-Ais Transfrontier Park and Tirasberg Conservancy.

Lastly, clades occurring in the Fynbos and Succulent Karoo (Ptr–Ptt-A and Ptt-D) generally exhibited low net diversification rates and high turn-over rates, particularly those in the Fynbos which that showed negative net diversification rates. These findings emphasize the importance of conserving the Fynbos and Succulent Karoo biomes.

## Conclusions

The study provided strong evidence that cladogenesis in *P. tentorius* can be linked to climatic fluctuations and topographic changes in Southern Africa since the Miocene, thus supporting our hypothesis. It appears if climate was of greater importance than topographic changes in earlier diversification events, but that uplift events together with climate change played a significant role in later divergences. The climatic and topographic changes linked here to early divergences in *P. tentorius* were also proposed for vicariance in other reptiles at the generic level. Ecologically, each clade in the northern group (Pv-B, Ptr and Pv-A) occurs in a unique niche shaped by different climatic factors. By contrast, clades in the southern group (Ptt-B, Ptt-C, Ptt-D and Ptt-A), showed no significant niche differences.

The results also correspond to other studies showing high taxon diversity (in terms of the number of clades) in the GCFR, not only for plants but also for animals, including reptiles. Diversification patterns of *P. tentorius* in the late Miocene and Pliocene thus seem to have paralleled those of other organisms, supporting the hypothesis of higher diversity in the GCFR than elsewhere over its distribution range. The strong association of *P. tentorius* clades with particular regions and vegetation types suggests that the clades evolved allopatrically and that contact in restricted areas is recent, following range expansions of some clades. However, although the clades abut, they do not necessarily overlap because vegetation in the regions regarded as possible intergradation zones forms a mosaic, which may still keep clades distinct.

Nevertheless, more research is necessary to establish if the clades hybridize in the so-called intergradation zones. Conservation awareness is warranted for all clades in the *P. tentorius* species complex, particularly for Ptt-B, Pv-B, Ptr, Ptt-A and Pv-A. Our study suggests that Ptt-B, Ptt-C, Ptt-A and Ptt-D are a single candidate species but with multiple conservation units, whilst, Ptr, Pv-A and Pv-B are three different species with different conservation units. This study together with the findings in [40] provides strong evidence that *P. tentorius* requires a taxonomic revision, which would impact the Red List Assessment of the species. As a species, the IUCN currently lists *P. tentorius* as “Near Threatened” [95] and *P. t. trimeni* as “Endangered” [95].

## Methods

### Taxon sampling

We sampled 404 specimens of *P. tentorius* from 76 localities throughout its distribution range in Southern Africa (see Additional file 5: Table S11), ensuring comprehensive coverage of its distribution range according to the literature [22, 37, 38, 51, 96, 97]. Collecting permits: Northern Cape Province (Permits No: FAUNA 1061/2/2015, FAUNA 1266/2016, FAUNA 1458/2015, FAUNA 1267/2016, FAUNA 0729/2018, FAUNA 0730/2018), Western Cape Province (Permit No: AAA007–00,179–0056) and Eastern Cape Province (Permits No: CRO 171/16CR, CRO 172/16CR), as well as the Ministry of Environment and Tourism, Namibia (Permit No: 1430/2009) were fully obtained.

### DNA extraction, PCR amplification and sequencing

Tortoises evolve slowly compared to most other reptiles [98]. Thus, fast evolving mtDNA markers are more suitable than slow evolving nDNA markers in reconstructing their phylogenetic histories, particularly those of small-scale species complexes [99, 100]. Townsend et al. [101] and Portik et al. [102] found that *PRLR* was one the fastest evolving nDNA maker for inferring phylogenies in the order Squamata. This study therefore used six mtDNA markers to generate the DNA sequence dataset: *12S rRNA* (*12S*, [103]), *16S rRNA* (*16S*, [104]), *Cytochrome b* (*Cyt-b*, [103, 105, 106], *NADH dehydrogenase subunit 4* (*ND4*, [107]), and the two *ND4* adjacent tRNA genes, *tRNA-His* and *tRNA-Ser* [107], and one fast evolving nDNA locus Prolactin Receptor Coding gene (*PRLR*, [101]).

The details of the DNA extraction, PCR amplification and sequencing are given in the Additional files. All NCBI GenBank accession numbers of sequence data are given in Additional file 5: Table S11. The optimal annealing temperatures and primers used for all markers are given in Additional file 6: Table S12.

All sequences were analysed using ABI Prism Sequencing Analysis software v.3.7 (Applied Biosystems), aligned with MUSCLE v.3.2 [108], gap open: − 400, gap extend: 0, clustering method: UPGMB, and minimum diagnostic length: 24. Indels and stop codons were manually checked with MEGA v.7 [109]. We employed PartitionFinder v.2 [110] under Python v.2.7 [111] to determine the best partition scheme for the concatenated dataset. We also used jModeltest v.2 [112] to determine the best substitution model and parameter settings for each data partition via AIC criterion (Additional file 1: Table S13). Nucleotide base biases across different partitions were determined through the homogeneity test implemented in PAUP v.4.0 [113]. Substitution saturation tests were performed using DAMBE v.6.1.9 [114] at both gene and partition (on protein coding gene) levels and were visually plotted for transitions and transversions against GTR-model modified genetic distance diagrams. This was done to investigate potential substitution saturation, particularly at the third codon position in the protein coding fast-evolving mtDNA. The DAMBE v.6.1.9 software was used to read sequence frames in order to determine the codon positions.

### Microsatellite DNA genotyping

Extracted genomic DNA was used as template DNA in the PCR. Twenty two microsatellite DNA loci, which were successful in previous testudines population genetics studies [115–121] were tested. They were tested on the target animal using PCR, with all forward primers labelled fluorescently with different dye probes (primer details and optimal annealing temperatures are given in Additional file 7: Table S14). All PCR reactions were performed using KAPA2G Robust HotStart Readymix, USA, in a BIO-RAD T 100™ Thermal Cycler (Singapore) under the following parameters: an initial 5 min denaturation step at 94 ºC, followed by 35 cycles of 30 s of denaturation at 94 ºC, 30 s of annealing (different optimal annealing temperatures were used depending on the loci, details given in Additional file 1: Table S14), and a 1 min. extension step at 72 ºC, with a final 10 min extension step at 72 ºC. The PCR annealing temperatures for the different microsatellite DNA loci were optimized using temperature gradient tests. Only loci showing a positive amplicon peak were used for further genotyping. For each PCR reaction, a 12.5 μl reaction was performed that contained 6.25 μl of the HotStart ReadyMix, 0.625 μl of a 10 mM forward primer, 0.625 μl of a 10 mM reverse primer, 3 μl of Millipore water, and 2 μl of template DNA with concentration of ~ 25 ng/μl.

We applied multiple mixed PCR’s to enable the simultaneous amplification of multiple microsatellite DNA markers. We subdivided the microsatellite markers into six multiple mixed PCR groups according to the type of dye probes, fragment lengths and optimal PCR annealing temperatures, to maximize visualization of the genotyping and to minimize the interference among the different loci during genotyping (details given in Additional file 7: Table S14). The PCR conditions, reagent concentrations for these six multiple mix reactions were the same as above for the single locus based PCR. The annealing temperature for each multiple mix reaction is also given in Additional file 7: Table S14.

In order to verify the microsatellite repeat motif unit in the *P. tentorius* species complex, and to ensure that the amplicons obtained from the dye labelled primer pairs indeed came from the target region, and not from other regions (caused by unspecific primer binding sites), we sequenced some individuals of the seven clades with the same primer pairs but without the dye labels (with the same PCR conditions as the above genotyping). The PCR products were electrophoresed in 1% agarose gel, visualized under UV light, and purified using a BioFlux PCR Purification Kit (Bioer Technology, China). Purified PCR products were cycle sequenced using BigDye (ABI PRISM® BigDye Terminator v3.1 Cycle Sequencing Kits, USA) and standard methods. The Big-Dye PCR products were purified with Zymo DNA Sequencing clean-up kits (Epigenetics Company, USA), prior to sequencing in an ABI 3500 genetic analyser.

We used PCR amplicon length for microsatellite genotyping and analyses throughout the study. In all cases, CONVERT [122] and PGDSpider [123] was used to convert datasets into different input file formats for different analyses. Sanger sequences were analysed using ABI Prism Sequencing Analysis software v.3.7 (Applied Biosystems), then aligned with MUSCLE v.3.2, gap open: − 400, gap extend: 0, clustering method: UPGMB, and minimum diagnostic length: 24. Indels and stop codons were manually checked with MEGA v.7. Microsatellite DNA was genotyped with GeneMarker v.2.4 [124].

Among these tested 22 microsatellite loci, 19 showed positive results and were used for carrying out further analyses. To ensure that the microsatellite dataset did not contain null alleles, stuttering or large allele dropout [125], we checked it using Micro-Checker v.2.2.3 [125] to avoid misleading genotyping results. For all classes a combined probability of *p* < 0.05 was considered a significant indicator of the presence of null alleles. Consequently, 14 out of the 19 loci were detected as negative in exhibiting null allele signal, and were used in further analyses (for details, see Additional file 8: Table S15).

### Genetic structure and phylogenetic analysis

To determine genetic structure and identify putative clusters in the microsatellite dataset, a Discriminant Analysis of Principal components (DAPC, [126]) was performed using the R package ‘adegenet’ [127] of the program R v 3.5.1 [128]. The optimal group membership cluster scheme was mapped using the “find, clusters” function. We used the BIC criterion to determine the optimal clustering scheme K. To determine the proper number of Principal Components (PCs) retained in the DAPC analysis and plot a scatterplot based on the reasonable number of PCs, we performed a cross–validation test using 300 PCs. Additionally, STRUCTURE v 2.3 [129] was used to map the best cluster scheme by using Admixture as ancestry model to allow individuals the possibility of having mixed ancestry with the Bayesian algorithms. In order to test genetic structure at different levels, we partitioned the microsatellite dataset into two testing schemes, and run STRUCTURE analysis on each schemes independently. In the first scheme, individuals were divided by the seven phylogenetic clades according to Zhao et al. [40], thus giving a total of seven populations. In the second scheme individuals were divided into 10 populations. Ptt-B was subdivided into a western and an eastern population. Pv-A was also subdivided into a western and eastern population. Both Ptt-B and Pv-A showed clear subdivisions in [40]. As for Pv-B, the northern population was morphologically distinct from the southern population, we therefore subdivided it into a northern and southern population, although the mtDNA loci did not show them as clearly separate according to Zhao et al. [40]. Analyses were run for 5 million generations using MCMC with the first 50, 000 discarded as burn-in. STRUCTURE HARVESTER [130] was used to summarize outputs of STRUCTURE. We used the optimal *ΔK* (based on In [Pr(X|K)] method described by Evanno et al. [50] to determine the most likely clustering scheme. The program Clumpak [131] was used to visualize population structure across multiple cluster schemes (clustering under different K values).

To compare the genetic structures between the microsatellite and sequence DNA datasets (mtDNA + nDNA), we first removed all sequences from alignments having outstanding microsatellite genotyping data to ensure that the mtDNA dataset completely matched the microsatellite DNA dataset. We then combined the trimmed mtDNA dataset with the nDNA dataset. We used BEAST v 2.4 [132] and the STACEY package [133], respectively, to carry out a Bayesian multispecies coalescent (MSC) phylogenetic analysis and to generate a species tree and a gene tree. We optimized the prior setting based on results of PartitionFinder and jModeltest (for details, see Additional file 1: Table S13). All individuals were assigned to the seven phylogenetic clades. We used a Yule prior and a relaxed log-normal clock. Trees and the relaxed log-normal clock model of all partitions were linked, while substitution models and parameters were unlinked. Analyses ran with 55 million generations, sampling every 5000 generations and discarding the first 25% as burn-in. Results were loaded into Tracer v 1.6 [134] to check sample mixing to ensure the majority of the parameters had Effective Sample Size (ESS) > 200. Both gene and species trees generated from BI analyses were imported into the TreeAnnotator package implemented in BEAST, and the maximum clade credibility trees with common ancestor heights were generated after the first 10% of trees was discarded as burn-in. In addition, we also performed a maximum likelihood analysis (ML) on the combined dataset using RAxML v.8 [135]. To evaluate the strength of support for each node, we used the same partitioning scheme as with the BEAST analysis, with 1000 nonparametric bootstrap replications with the rapid-hill climbing algorithm [136]. The number of alternative runs on distinct starting trees was 100. The ML algorithm model selected was GTRCAT. In all cases, bootstrap support (BP) > 70% for ML [137] and posterior probability (PP) > 0.95 for Bayesian inference [138] were considered as strongly supported.

To test whether Fynbos and Succulent Karoo vegetation of the GCFR had greater diversity than other biomes, we used ArcGIS v.10.4 (ESRI, 2016) to strictly examine the biome of each individual on the basis of genetic structure. We then counted the taxon diversity in the Fynbos, Succulent Karoo and Nama Karoo.

Lastly, to test the degree of gene flow and isolation among the seven clades, and to determine whether the seven clades were truly isolated, we used Migrate v 3.2 [139, 140] analysis with our microsatellite dataset. During the analysis, we estimated the pairwise migration rates among the seven mtDNA clades with a long MCMC chain, sampling every 1000 steps for a total of 100 million genealogies after a burn-in of 100,000 steps. We used the Brownian motion stepwise mutation model in the analysis. The Bayesian estimation of population size and migration rates was run with static heating (1, 1.5, 3.0, 10 000 000) and one long chain under a full model with all migration rates and population sizes. The parameters M and Θ were both generated from *F*_*ST*_–calculations during the analysis.

### Divergence time dating

In order to generate an ultrametric tree to meet the requirements of habitat reconstruction analysis, we used DnaSP v.5 [141] to determine the haplotype sequences, and removed all the excess identical sequences in the concatenated mtDNA and nDNA datasets. Since the saturation test did not detect nucleotide saturation, we performed the complete dating analyses with the combined mtDNA + nDNA and mtDNA sequence datasets, respectively. We used 171 haplotype sequences to perform the Bayesian calibration dating analysis with the program BEAST v.2.4.8 with StarBEAST package for both the combined and mtDNA datasets. The priori setting for both analyses were designated based on the seven phylogenetic clades supported by our phylogenetic analyses (both mtDNA and the combined dataset). Each partition was applied with different optimized substitution models and parameters determined by another independent test using PartitionFinders v.2 and jModeltest v.2 (see Additional file 1: Table S16). Trees and clock models were linked, site models unlinked, and dating priors in the most recent common ancestor (MRCA) set based on five calibration nodes from a dating study using the complete mtDNA genome [142]. For the mtDNA dating analysis, the log normal MRCA prior setting details are given in Additional file 1: Table S17. Standard deviation was set at 5% of the age value of each constrained node, and the offset was set as the same age value of each constrained node was allocated to each constrained node for calibration. For the mtDNA + nDNA based dating analysis, we specified log normal MRCA based on a range between the dating results from Kehlmaier et al. [142] and Hofmeyr et al. [36] (details in MRCA prior settings given in Additional file 1: Table S17), again, standard deviation was set at 5% of the lower age limit value of each constrained node, and the offset was used the same value of the lower age limit on each constrained node were weighted on each MRCA. For both dating analyses, a sampling gap between zero and two for each log normal MRCA and a log normal relaxed clock model were specified. The MCMC chain lengths were set as 55 million generations to enable the majority of parameters to be well sampled (with ESS > 200), sampling every 5000 chains. Results from each analysis were inspected with Tracer to check standard deviations and sampling adequacy (ESS > 200). For both analyses, we used Yule as Bayesian inference model. For both analyses, the gene and species trees generated from the BI analyses were imported into the TreeAnnotator package implemented in BEAST, and the maximum clade credibility tree with common ancestor heights generated after the first 10% of trees was discarded as burn-in.

### Spatial AMOVA to define geographic group structure

In order to define population groups that were geographically homogeneous and maximally differentiated from each other, as well as to identify the potentially crucial genetic barriers that separate these population groups, we used SAMOVA 2.0 [143] with concatenated mtDNA to delineate groups with temporal and spatial dimensions. SAMOVA analyses with ranges of K = 5 to K = 8 were tested, and the results with minimal subdivision in clades were used for further habitat reconstruction analysis.

### Habitat reconstruction analysis

This analysis was done to test how the shifting of geographic regions, biomes and topographical barriers (GE and CFMs) influenced diversification process against a timeline. Three independent habitat reconstruction analyses were performed on the seven geographic regions, the three biomes and the three regions separated by the topographical barriers specified below.

First, we partitioned the distribution range of the *P. tentorius* species complex into seven main geographical areas according to the optimal grouping scheme suggested by results of the SAMOVA 2.0 analysis (see Fig. 1). Second, we split its range into the three biomes, namely, Nama Karoo, Succulent Karoo, and Fynbos. Last, we partitioned its range into three regions: north of the GE, between the GE and CFMs, and south of the CFMs. Biogeographic reconstructions were then performed on each of these three partitions with the R package BioGeoBEARS [144–146] via the program RASP v.4.0 [147], respectively. To unify the methods of biogeographical inference, such as the dispersal-vicariance analysis (DIVA), the Dispersal-Extinction-Cladogenesis (DEC-Lagrange) analysis and the Statistical BayArea analysis, the BioGeoBEARS analysis first tests the optimal biogeographic reconstruction model by evaluating the founder effect parameter “J” [148, 149]. Thus, for each of the three analyses, we first compared combinations of the three models with and without “J”, and selected the optimal model based on AIC corrected (AICc) and AIC weight criteria with the LRT test. The ultrametric species tree generated by BEAST BI analysis with the mtDNA + nDNA dataset was used for all three biogeographic reconstruction analyses. All outgroups were truncated before initiating analyses to improve the accuracy of the reconstruction results [147].

### Diversification rate analysis

In order to detect and visualize the diversification dynamics, evolution rate heterogeneity and significant rate shifts across different clades on the calibrated BEAST chronogram (mtDNA + nDNA dataset), we employed the Bayesian statistical framework approach [150, 151] of the BAMM software [152], together with the R packages ‘BAMMtools’ [152] and Ape [153]. We used the “setBAMMpriors” function to determine a proper prior for the expected number of shifts, initial λ, λ shift and initial μ. Analyses were conducted with four simultaneous MCMC chains for 50 million generations, using the “speciation–extinction” model, deltaT = 0.1, Swap period = 1000 and default settings for the rest of the parameters. Pairwise probability comparisons obtained were then used to reconstruct a matrix with macroevolutionary cohort analysis [154] to look for differences in the macroevolutionary rate regime among the lineages of the *P. tentorius* species complex based on haplotypes. A phylorate plot with optimal rate shift scheme was generated through BAMMtools to visualize evolutionary rate variation across the Bayesian phylogenetic tree topology. Rate through time (RTT) plots were constructed using BAMM on all groups to visualize the diversification rate shift against temporal scales in different groups. To compute the diversification rate (λ) and extinction rate (μ) independently for each clade and crucial evolutionary group, R packages ‘dplyr’ [155], ‘readr’ [156] and ‘ggplot2′ [157] were used to calculate λ and μ for each target group. The net diversification (r = λ – μ) and turn-over rates (t = μ /λ) were used as indicators to evaluate the diversification of the *P. tentorius* species complex against temporal dimensions [158], as well as predict future trends.

In addition, to validate the diversification rate results estimated from BAMM and to evaluate the robustness of the diversification pattern against time as predicted by the BAMM analyses, we performed the TESS Bayesian inference analysis [159], using the R package ‘TESS’ [159]. TESS is a powerful analytical tool for inferring rates of lineage diversification from empirical phylogenetic trees under various stochastic branching process models [159]. It can also be used to detect mass extinction events based on the computation of Bayes factors. For details on parameter and prior settings of running TESS analysis, see Additional files.

### Character dependency analysis

A character dependency analysis was used to verify whether the diversification of the *P. tentorius* species complex was influenced statistically by geographic barriers and biomes. When considering the OR as a geographic barrier, we used the BiSSE approach [160] instead of the GeoSSE approach [161] because there is no clear evidence of population overlap across the OR. We performed the BiSSE analysis (6 parameters) with R packages ‘ape’, ‘diversitree’ [162] and ‘phytools’ [163] to investigate whether the OR significantly influenced the cladogenic history of the *P. tentorius* species complex. For details on running BiSSE analysis, see Additional files.

We used the MuSSE approach [164] implemented in R packages ‘ape’, ‘diversitree’, ‘phytools’ and ‘geiger’ [165] to determine whether barriers like the GE and Swartberg Mountain (SM) significantly influenced the diversification of the *P. tentorius* species complex. We partitioned the complex’s distribution range into three geographic regions as “1” (above the GE), “2” (below the GE, but north of the SM including the western region below the GE) and “3” (south of the SM). In total, 12 parameters were included in the analysis. For details on running MuSSE analysis, see Additional files.

The same method was used to model the interactions between cladogenesis and biome. To simplify the biome analysis and interpretation, we excluded Albany Thicket and included only Nama Karoo, Succulent Karoo and Fynbos. Furthermore, we considered only Succulent Karoo as relevant in the Little Karoo because tent tortoises prefer this vegetation type in that region. Our biome assessment included 1) Nama Karoo on the northern and southern sides of the GE, 2) Fynbos on the western side of the GE and 3) Succulent Karoo along the west coast and in the Little Karoo, and we encoded them as characters “1”, “2” and “3”, respectively. The rest of the steps followed the MuSSE analysis of the geographic regions. For all the above BiSSE and MuSSE analyses, the input tree used was the calibrated BEAST chronogram with the mtDNA + nDNA dataset.

### Environment niche modelling analysis

To accurately estimate suitable habitats through timelines (from past, current to future), 455 samples with genetic information confirmed were used. We did not include museum or internet records which lacked genetic information, since many of the museum collections have problems of incomplete information. Datasets with detailed coordinate information were partitioned into seven separate datasets, based on the seven clades. In total, eight datasets were independently analysed by ENM, namely, datasets [*P. t. tentorius* (Ptt-A – Ptt-D), *P. t. trimeni* (Ptr), *P. t. verroxii* (Pv-A and Pv-B)] and the combined dataset (including all 455 specimens of *P. tentorius* used in this study). Each dataset was written in WGS1984 pseudo-projection format. To reduce the sampling bias and spatial auto-correlation, we used the R package ‘spThin’ [166] to thin each dataset to no more than one sample per kilometre.

We downloaded 19 bio-climatic variables (2.5′ grid, Bio1–19, see Additional file 9: Table S18) that simulated Community Climate System Model 4 (CCSM4), from the WorldClim 1.4 platform [167] for five different time periods: Last Interglacial (LIG, ranging from 120–140 thousand years ago), Last Glacial Maximum (LGM, 22 thousand years ago), Middle Holocene (MIDH, 6 thousand years ago), Current (1960–1990 AD) and Future (average for 2061–2080 AD). All climatic raster layers were clipped to our target region (Longitude: 10° E – 38° E, Latitude: − 38° S – − 20° S) via QGIS v. 2.18.24 (QGIS Development Team, 2014). Each raster layers was set to the same spatial extent and resolution with the R package ‘raster’ [168].

To decrease the correlation between the bio-climatic variables, we first performed a preliminary ENM analysis with all 19 variables. One hundred bootstrap replications were run in the program Maxent v 3.4.1 [169], with 10,000 maximum iterations. A jack-knife method was applied to measure variable importance. The R packages ‘maptools’ [170], ‘rgeos’ [171], ‘raster’, ‘rgdal’ [172] and ‘dismo’ [173] were used to calculate the pairwise correlation of the 19 variables. If the correlation coefficient between two variables was greater than 0.7, we omitted the one with the lower contribution based on the average contribution from the preliminary ENM run. After the omission procedure of the correlation test, we retained seven variables: Bio1, Bio3, Bio4, Bio8, Bio9, Bio12 and Bio19 (Additional file 10: Table S19) to estimate the potentially suitable habitats for each clade and the entire *P. tentorius* species complex across the timeline.

To optimize the parameter settings, the R package ‘ENMeval’ [174] was used to estimate the parameter settings for Linear features, Quadratic features, Product features, Hinge features and the Regularization multiplier. For the datasets with fewer than 50 samples, we used the “leave-one-out” method for specifying cross-validation with the model folds = sample size; whilst, for datasets with 50 or more samples, we use the cross-validation with 10 model folds. After parameter optimization, each dataset was ran with Maxent. For all datasets, the area under the ROC curve (the AUC criterion) was used as indicator to evaluate the performance of the ENM analyses. A threshold with AUC above 0.75 was considered as a robust prediction [46]. The AUC results of each dataset is given in Additional file 1: Table S6. To calculate the area of suitable ranges, the average final outputs in logistic format of each dataset was reclassified into five different classes, ranging from 0 to 1, “0” indicating not suitable, “1” indicating most suitable, each class with 0.2 as interval, using ArcGIS v10.4 (ESRI, 2016). The “raster calculator” implemented in ArcGIS was used to calculate area (with threshold > 0.2).

Moreover, to determine if the niches between two species, as calculated by Maxent, were significantly different, we used the same raster files as in the ENM analyses and the average final outputs generated with the ENM analyses, as input file in the program ENMtools [175]. The I statistic approach was used as metric during each run [47]. In this analysis, we used only the current dataset of each data partition. The results generated from ENMtools were summarized and visualized in R v 3.5.1 [128].

## Supplementary information

**Additional file 1: Figure S1.** The results of the Bayesian clustering analysis with STRUCTURE. The plot shows the values of ΔK calculated according to Evanno et al. [47].** Figure S2.** (a) Results of BIC value versus the number of clusters to determine the optimal clustering scheme in the DAPC analyses. (b) The Cross-Validation test results determining the optimal number of PCs retained. **Figure S3.** The species tree chronograms generated from the BEAST calibration dating analyses, A: mtDNA chronogram, B: mtDNA+nDNA chronogram. The red dots were the five constrained calibration points for calibration dating analyses. **Figure S4.** The macroevolution cohort matrix for the seven clades of the P. tentorius species complex. BAMM Bayesian diversification rate analysis based on the mean phylorate plot trees, are shown at the top and on the left side of the cohort matrix, for purposes of comparison. The matrix shows pairwise probabilities of two groups sharing the same evolutionary dynamics. The “warm” colours represent high cohort similarities (highest value “1” refers to 100% similarity), whilst, the “cool” colours represent low cohort similarities (lowest value “0” refers to 0% similarity). **Figure S5.** Visualizing the single-chain MCMC diagnostics for a CoMET analysis with empirically estimated diversification hyperpriors. Blue bars/dots represent passed tests and red bars/dots mean failed tests (failed convergence). **Table S3.** The node age (Ma) and 95% HPD at each node (see Fig. 5 and Fig. S3) generated from BEAST calibration dating analyses in both gene trees and species trees of the mtDNA and (mtDNA+nDNA) datasets, respectively. **Table S4.** The independent BioGeoBEARS model test results for the six habitat reconstruction models with consideration of the “founder effect” parameter “J” on geographic regions, biome and topographic barriers datasets. The selected best model of each analysis with its criteria is shown in bold. **Table S5.** The ANOVA based LRT test results retrieved from the likelihood function models of different scenarios investigated under character dependency analyses BiSSE and MuSSE with different models of regions, biomes and the two sides of the Orange River. “Minimal”: the null model assumed all parameters are equal between different characters states; “all different”: the full parameter model considered lambda, mu and q as different between character states; “free.lambda”: the model considered lambda as different only between different character states, “free.mu”: the model considered only mu as different between different character states, “free.lambda.mu”: the model considered only lambda and mu as differ between different character states, “free.q”: the model considered only q as different between different character states. The best model with its criteria from the LRT test in each analysis is shown in bold. **Table S6.** The average test AUC criterion under the ROC curve and its standard deviation (SD) generated by ENM analyses for each group [P. t. tentorius (Ptt-A – Ptt-D), P. t. trimeni (Ptr), P. t. verroxii (Pv-A and Pv-B), and the entire P. tentorius species complex] through the given timeline. LIG: Last Interglacial, LGM: Last Glacial Maximum, and MIDH: Middle Holocene. **Table S7.** The results of the optimization of parameters settings for all ENM analyses across groups (group abbreviations as in Table S6, with All denoting the entire P. tentorius species complex) and timelines. Features: Linear features (L), Quadratic features (Q), Product features (P) and Hinge features (H); RM = Regularization multiplier. LIG: Last Interglacial, LGM: Last Glacial Maximum, and MIDH: Middle Holocene. **Table S8.** The pairwise results of niche difference analysis between clades (taxon abbreviations the same as group abbreviations in Table S6). The pairwise comparisons showing significant niche differences are in bold, whilst, comparisons without significant niche differences are underlined. Note: If the point estimated value (the matrix at the bottom-left) was lower than the 5% threshold value (the matrix at the top-right), then the niches were significantly different. **Table S9.** The ENM analyses estimating the area of suitable habitats of the seven clades (group abbreviations as in Table S6), as well as changes in the area of suitable habitats against the timeline (for details about the timeline see the Materials and Methods). The arrow " ↑ " represents increasing area, whilst, " ↓ " indicates decreasing area. Groups currently showing a decrease in the area of suitable habitats or future habitats are given in bold. **Table S13.** Optimal partition scheme, substitution model, likelihood score (-InL), Gamma shape, proportion of estimated invariable sites for the mtDNA+nDNA based BEAST phylogenetic inference. **Table S16.** Optimal partition scheme, substitution model, likelihood score (-InL), Gamma shap, and proportion of estimated invariant sites for the BEAST calibration dating analyses. **Table S17.** The constraint of the five most recent common ancestors used in the BEAST calibration dating analyses for the mtDNA and mtDNA+nDNA datasets.

**Additional file 2: Table S1.** The sequence polymorphisms of all genes used in this study, including fragment length (bp), number of variable sites, variable site percentage, parsimony informative sites, and the percentage of parsimony informative sites.

**Additional file 3: Table S2.** The Migrate analysis results (using microsatellite data) estimating effective population size of the seven mtDNA clades (Θ1-Θ7). Population 1 represents Ptt-B, population 2 represents Pv-B, population 3 represents Ptr, population 4 represents Ptt-C, population 5 represents Ptt-A, population 6 represents Pv-A, and population 7 represents Ptt-D. In terms of potential gene flow rate (M), M1->2 denotes the gene flow rate from Ptt-B to Pv-B. The directional gene flow occurring in the two intergradation zones, between Ptt-B and Pv-B, and between Pv-B and Ptt-C is in bold.

**Additional file 4: Table S10.** The climatic variables that made significant impacts in each group across different periods (group abbreviations as in Table S6) from the ENM analyses.

**Additional file 5: Table S11.** List of all samples, their corresponding localities and NCBI GenBank accession numbers across different genes. All NCBI accessions of outgroups used in this study are given at the bottom.

**Additional file 6: Table S12.** List of primers used in the study with corresponding oligo sequences, optimized annealing temperatures and sources.

**Additional file 7: Table S14.** Allele size ranges, repeat motifs with their NCBI GenBank accession numbers, multiple-mix reaction grouping schemes, the oligo-nucleotide sequences of primers with dyes, optimal annealing temperatures and the sources of primers of all microsatellite DNA markers tested in this study. "NA" indicates the primer pair failed to amplify.

**Additional file 8: Table S15.** The genotyping results of 14 microsatellite DNA loci used in this study, together with locality and subpopulation information.

**Additional file 9: Table S18.** The bioclimatic variables and their percentage contribution, permutation importance and jack-knife test AUC’s used in the ENM to determine suitable habitat ranges of the P. tentorius species complex. Variables with AUC above 0.75 were considered as potentially useful (given in bold).

**Additional file 10: Table S19.** The correlation matrix retrieved from the correlation test between each pair of bioclimatic variables. The R-square values varied from zero to one, with "zero" representing no correlation and "one" representing 100% correlation. The bioclimatic variables (in bold) are the ones used in further niche modelling analyses after trimming variables showing strong correlation. The pairwise values (R-square) > 70 or < -70 were considered as strong correlations. All pairwise R-square values showing a strong correlation between bioclimatic variables are underlined.

## Data Availability

The data supporting the conclusions of this article are included in the Additional files of the article. The datasets/alignments, trees with the tip labels, are also included in the Additional files.

## Abbreviations

Ptt-A: *Psammobates tentorius tentorius*-Clade A
Ptt-B: *Psammobates tentorius tentorius*-Clade B
Ptt-C: *Psammobates tentorius tentorius*-Clade C
Ptt-D: *Psammobates tentorius tentorius*-Clade D
Pv-A: *Psammobates tentorius verroxii*-Clade A
Pv-B: *Psammobates tentorius verroxii*-Clade B
Ptr: *Psammobates tentorius trimeni*
CFR: Cape Floristic Region
OR: Orange River
GCFR: Greater Cape Floristic Region
CFM: Cape Fold Mountain
GE: Great escarpment
ENM: Ecological Niche Modelling
ML: Maximum likelihood
BI: Bayesian inference
BP: Bootstrap
PP: Posterior probability
