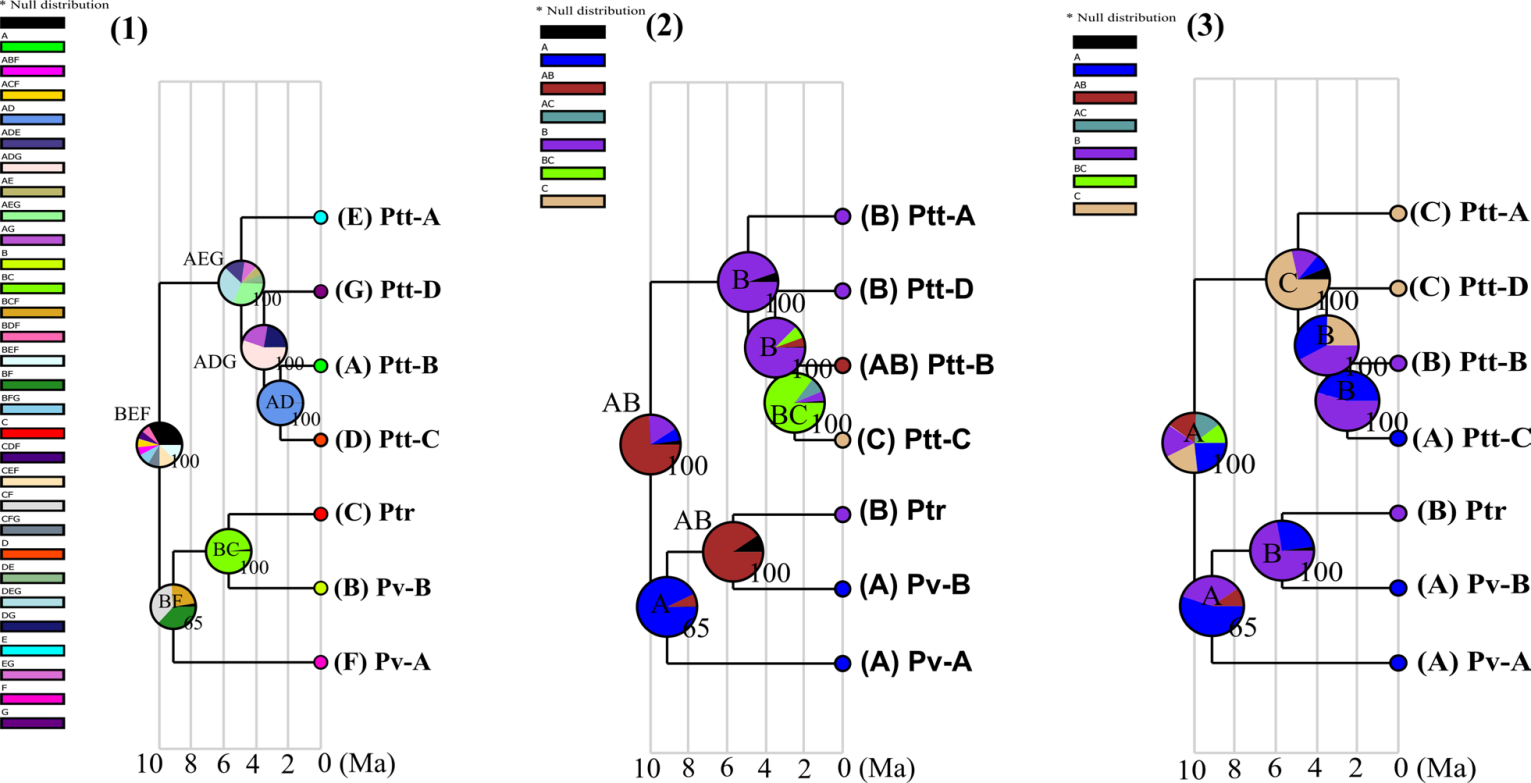# Correction to: Climatic and topographic changes since the Miocene influenced the diversification and biogeography of the tent tortoise (*Psammobates tentorius*) species complex in Southern Africa

**DOI:** 10.1186/s12862-021-01868-9

**Published:** 2021-07-09

**Authors:** Zhongning Zhao, Neil Heideman, Phillip Bester, Adriaan Jordaan, Margaretha D. Hofmeyr

**Affiliations:** 1 Department of Zoology and Entomology, University of the Free State, Biology Building B19, 205 Nelson Mandela Dr, Park West, Bloemfontein, South Africa; 2 Department of Virology, University of the Free State and National Health Laboratory Service (NHLS), Bloemfontein, South Africa; 3 Department of Biodiversity and Conservation Biology, Chelonian Biodiversity and Conservation, University of the Western Cape, Bellville, South Africa

## Correction to: BMC Evol Biol (2020) 21:271 10.1186/s12862-020-01717-1

Following the publication of the original article [1], the authors notified us of the following errors which need correcting:

1. In the paragraph: “*Psammobates tentorius* dispersed to the southern region of South Africa by the early Pliocene. The first divergence at node n7 isolated Ptt-A in the western Little Karoo from the rest of *P. t. tentorius*…..”, the highlighted part in the sentence following it, “Thereafter, Ptt-D in the eastern Little Karoo Oudtshoorn basin diverged from *P. t. tentorius* clades 1 and 4,” should be replaced so that it reads as follows “Thereafter, Ptt-D in the eastern Little Karoo Oudtshoorn basin diverged from *P. t. tentorius* clades Ptt-B and Ptt-C”. The oversight arose when changing to the new clade names as suggested by the reviewers.
2. The caption of Fig. 5 was erroneously presented (in terms of the arrangement of the words) in the online version of the article. For some reason, the part “The chronograms from the concatenated dataset generated from the BEAST Bayesian calibration dating analyses with a background temperature fluctuation diagram” moved to the back of the caption.

**The correct version should read:**

Figure 5. The chronograms from the concatenated dataset generated from the BEAST Bayesian calibration dating analyses with a background temperature fluctuation diagram (modified from Zachos et al. 2001 and Zachos et al. 2008). (a) The mtDNA based dating results, (b) the mtDNA + nDNA based dating results. The interval of the diversification of* P. tentorius* highlighted in blue colour represents the re-establishment of major ice sheets due to cooling. The red spots symbolize the five calibration nodes from the literature used for the calibration dating analyses. The three grouping schemes were the following. (1) The seven geographic regions defined by the SAMOVA analysis and indicated as “A–G”. (2) The distribution of the seven clades according to biomes, “1” = Nama Karoo, “2” = Succulent Karoo and “3” = Fynbos. (3) The regions separated by the critical barriers, “1” = north of the Great Escarpment (GE), “2” = region between the GE and Swartberg Mountain (SM), “3” = south of the SM. The sample size of each clade was also given. Nodes n1–n9 represent the divergence events that the cladogenic diversification of the genus* Psammobates* involved. The posterior probabilities are indicated at each node.

3. We also noticed that the original colour scheme of Fig. 3 was changed during production. It resulted in some clusters in* P. t. verroxii*—group B, not being distinguishable from some clusters in *P. t. tentorius*—group B, particularly for the cluster coloured in “chartreuse”. This problem is not visible in the PDF version, but only in the online version. The correct Fig. 3 (with the correct colour scheme) is attached to this correction.
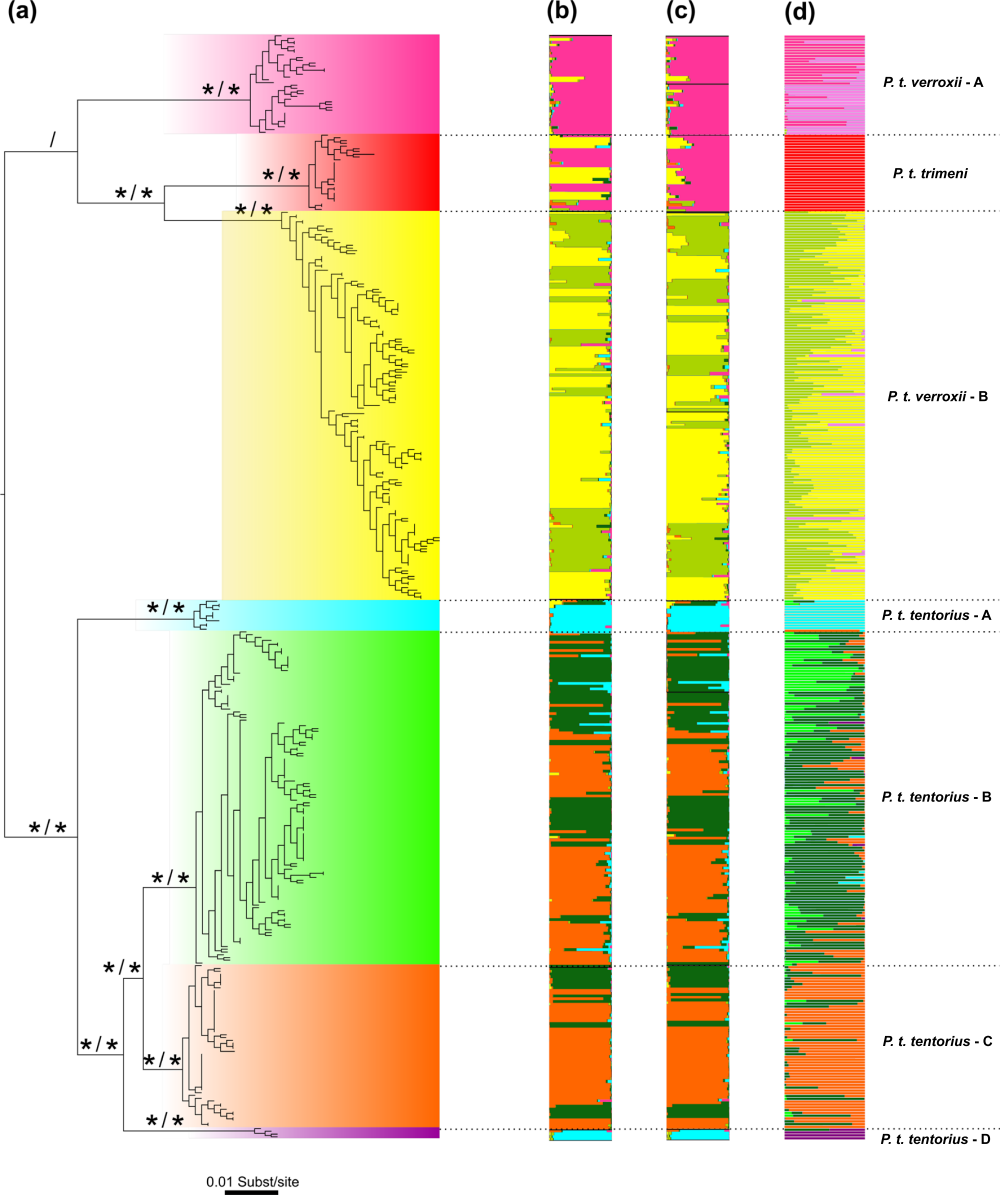
4. Part of the caption in Fig. 3 was incorrectly arranged. In the sentence “The clustering results from the STRUCTURE analyses (K = 6) based on b sub-populations, and c on clades. d DAPC analysis with 14 microsatellite DNA loci are shown to the right of the tree”. The highlighted part “b sub-populations, and c on clades” should be changed to “b clades, and c on sub-populations”.
5. In Fig. 6, the number on the timeline of the chronogram was rearranged during production of the online version. The correct Fig. 6 is also attached to this correction.