# Supplementary material for: Climatic and topographic changes since the Miocene influenced the diversification and biogeography of the tent tortoise (Psammobates tentorius) species complex in Southern Africa
Source: BMC Evol Biol. 2020 Nov 13;20:153. doi: 10.1186/s12862-020-01717-1 (PMC7666511; doi:10.1186/s12862-020-01717-1)
Supplement: Supplementary file 1 — Additional file 1: Figure S1. The results of the Bayesian clustering analysis with STRUCTURE. The plot shows the values of ΔK calculated according to Evanno et al. [47]. Figure S2. (a) Results of BIC value versus the number of clusters to determine the optimal clustering scheme in the DAPC analyses. (b) The Cross-Validation test results determining the optimal number of PCs retained. Figure S3. The species tree chronograms generated from the BEAST calibration dating analyses, A: mtDNA chronogram, B: mtDNA+nDNA chronogram. The red dots were the five constrained calibration points for calibration dating analyses. Figure S4. The macroevolution cohort matrix for the seven clades of the P. tentorius species complex. BAMM Bayesian diversification rate analysis based on the mean phylorate plot trees, are shown at the top and on the left side of the cohort matrix, for purposes of comparison. The matrix shows pairwise probabilities of two groups sharing the same evolutionary dynamics. The “warm” colours represent high cohort similarities (highest value “1” refers to 100% similarity), whilst, the “cool” colours represent low cohort similarities (lowest value “0” refers to 0% similarity). Figure S5. Visualizing the single-chain MCMC diagnostics for a CoMET analysis with empirically estimated diversification hyperpriors. Blue bars/dots represent passed tests and red bars/dots mean failed tests (failed convergence). Table S3. The node age (Ma) and 95% HPD at each node (see Fig. 5 and Fig. S3) generated from BEAST calibration dating analyses in both gene trees and species trees of the mtDNA and (mtDNA+nDNA) datasets, respectively. Table S4. The independent BioGeoBEARS model test results for the six habitat reconstruction models with consideration of the “founder effect” parameter “J” on geographic regions, biome and topographic barriers datasets. The selected best model of each analysis with its criteria is shown in bold. Table S5. The ANOVA based LRT test results retrieved from [file 12862_2020_1717_MOESM1_ESM.docx]

**Title: Climatic and topographic changes since the Miocene influenced the diversification and biogeography of the tent tortoise (*Psammobates tentorius*) species complex in Southern Africa**

**Authors**: Zhongning Zhao^1^, Neil Heideman^1,*^, Phillip Bester^2^, Adriaan Jordaan^1^ and Margaretha D. Hofmeyr^3^

**Supplementary Materials**

***DNA extraction, PCR amplification and sequencing***

We collected fresh tissue from tail tips (15–25 mg), tissue from dead carcasses or blood (0.1 ml) from the subclavian vein. Fresh tissue was preserved in 96% ethanol and blood was dissolved in 1% of a 10M Sodium-EDTA anticoagulant solution. Connective tissue and bone were kept dry in vials, whereas preserved muscle was stored in 96% ethanol. All samples were stored at –80 ºC in the laboratory until we did the DNA extraction. Genomic DNA was extracted using QIAGEN DNeasy Blood and Tissue kits (QIAGEN, Germany) following the manufacturer’s instructions. For the ancient DNA extraction from museum specimens or severely degraded shells, we followed the protocol used in [1].

Polymerase Chain Reactions (PCR) were performed in a BIO-RAD T 100^TM^ Thermal Cycler (Singapore) under the following parameters: an initial 4 min denaturation step at 94 ºC, followed by 37 cycles (43 for the museum specimens) of 30 s denaturation at 94 ºC, 30 s annealing (62 ºC for *12S*; 50 ºC for *16S*; 51 ºC for *Cyt-b*; 61 ºC for *ND4* with *tRNA-His* & *tRNA-Ser*; 58 ºC for *PRLR*), and 1 min extension at 72 ºC, with a final 10 min extension step at 72 ºC. PCRs were performed using KAPA2G Robust HotStart ReadyMix, USA. For each PCR reaction, a 12.5–μl reaction was performed that contained 6.25 μl of the HotStart ReadyMix, 0.625 μl of a 10 mM forward primer, 0.625 μl of a 10 mM reverse primer, 3 μl of Millipore water, and 2 μl of template DNA with concentration of ~25 ng/μl. The PCR products were electrophoresed in a 1% agarose gel, visualized under UV light, and purified using a BioFlux PCR Purification Kit (Bioer Technology, China). Purified PCR products were cycle sequenced using BigDye (ABI PRISM® BigDye Terminator v3.1 Cycle Sequencing Kits, USA) and standard methods, 3 μl of the purified PCR product, 4 μl of the fluorescent-dye terminators with an ABI PRISM Dye Terminator Cycle Sequencing Reaction Kit, PerkinElmer, and 3 μl of a 10 μM primer solution for each primer pair (with annealing temperatures of 60 ºC for *12S*; 50 ºC for *16S*; 50 ºC for *Cyt-b*; 57 ºC for *ND4* with *tRNA-His & tRNA-Ser*; 57 ºC for *PRLR*). The Big-Dye PCR products were purified using a Zymo DNA Sequencing clean-up kit (Epigenetics Company, USA), prior to sequencing in an ABI 3500 genetic analyser.

***TESS analysis***

To specify the prior distribution for speciation and extinction rate parameters can be challenging. The CoMET analysis of TESS allows implementing an automatic empirical hyperprior procedure which performs an initial Bayesian MCMC simulation analysis under a constant–rate birth–death process model to provide reasonable priors for hyperparameters of diversification. We used the same input tree as in the BAMM analysis (the BEAST chronogram of the mtDNA+nDNA dataset), and all outgroups were also pruned using RASP v.4.0 as in BAMM analysis. The CoMET analysis was run with “tess.analysis” function with maximum 300,000 interactions. We then summarized the analysis results and visualized the output using “tess.process.output” and “tess.plot.output” functions, respectively. The speciation rates (with rate shift frequency), extinction rates (with rate shift frequency) and mass extinction Bayes factor with frequency were plotted. Lastly, in order to ensure that reliable estimations were obtained from the MCMC simulation of CoMET analysis, we performed the MCMC diagnosis for the CoMET output using the Single–chain diagnostics method. During the MCMC diagnosis, we assessed convergence using two diagnostics: the effective sample size (ESS) and the Geweke statistic. As a rule of thumb, higher ESS values indicate more precise inferences from the posterior sampling, and the ESS should be larger than 200. For the Geweke statistic, we expect it to pick up a low level of non–convergence.

***BiSSE analysis***

To do BiSSE analysis, we coded populations north of the OR as the “0” state, and populations south of it as the “1” state. We then built likelihood functions into different scenarios: a) full model [(through function ‘all.different’, λ1 ≠ λ2, μ1 ≠ μ2, q01 (transition rate from character “0” to “1”) ≠ q10 (transition rate from character “1” to “0”)], b) λ different model (through function ‘free.λ’, λ1 ≠ λ2, μ1 = μ2 and q01 = q10), c) variable q model (through function ‘free.q’, λ1 = λ2, μ1 = μ2 and q01 ≠ q10). We used the ANOVA based likelihood ratio test (LRT) to determine the “best scenario” under the criteria ln likelihood value and AIC criterion. The best-fit model was used to run a Bayesian MCMC analysis. First a preliminary run with 100 steps was done to estimate parameter settings. The formal long run was done with 100,000 steps, sampling every 1000 steps.

***MuSSE analysis***

Different models were built, based on several scenarios as different likelihood functions: a) a “null” model (in function ‘Minimal’), which regarded all λ, μ and q to be equal across the different geographic regions (λ1 = λ2 = λ3, μ1 = μ2 = μ3 and q12 = q21 = q13 = q31 = q23 = q32), b) a “full” model (in function ‘all different’), which regarded all λ, μ and q to be different (λ1 ≠ λ2 ≠ λ3, μ1 ≠ μ2 ≠ μ3 and q12 ≠ q21 ≠ q13 ≠ q31 ≠ q23 ≠ q32), c) considered differences at λ only (by function ‘free.λ’, λ1 ≠ λ2 ≠ λ3, μ1 = μ2 = μ3 and q12 = q21 = q13 = q31 = q23 = q32), d) considered differences at μ only (in function ‘free. μ’, μ1 ≠ μ2 ≠ μ3, λ1 = λ2 = λ3 and q12 = q21 = q13 = q31 = q23 = q32), e) considered λ & μ to vary, but that character transitions are orderly (through function ‘free.λ. μ’, μ1 ≠ μ2 ≠ μ3, λ1 ≠ λ2 ≠ λ3 and q12 = q21 = q13 = q31 = q23 = q32), f) λ and μ are constant, but the transition process is fully flexible (through function ‘free.q’, λ1 = λ2 = λ3, μ1 = μ2 = μ3 and q12 ≠ q21 ≠ q13 ≠ q31 ≠ q23 ≠ q32). We then performed an ANOVA based LRT test to determine the most likely scenario. In addition, we used AIC weight criteria to select the best fitting model, which was used to run a Bayesian MCMC. Again, we made a preliminary run to determine prior settings. The formal long run was conducted with 100,000 steps, sampling every 1000 steps.

**Supplementary Tables**

*** Note**: Tables which were too large to fit in the main text and are therefore supplied separately as single Excel files.

**Table S1***. The sequence polymorphisms of all genes used in this study, including fragment length (bp), number of variable sites, variable site percentage, parsimony informative sites, and the percentage of parsimony informative sites.

**Table S2***. The Migrate analysis results (using microsatellite data) estimating effective population size of the seven mtDNA clades (Θ1-Θ7). Population 1 represents Ptt-B, population 2 represents Pv-B, population 3 represents Ptr, population 4 represents Ptt-C, population 5 represents Ptt-A, population 6 represents Pv-A, and population 7 represents Ptt-D. In terms of potential gene flow rate (M), M1->2 denotes the gene flow rate from Ptt-B to Pv-B. The directional gene flow occurring in the two intergradation zones, between Ptt-B and Pv-B, and between Pv-B and Ptt-C is in bold.

**Table S3**. The node age (Ma) and 95% HPD at each node (see Fig. 5 and Fig. S3) generated from BEAST calibration dating analyses in both gene trees and species trees of the mtDNA and (mtDNA+nDNA) datasets, respectively.

|  |  |  |  |  |  |  |  |  |
| --- | --- | --- | --- | --- | --- | --- | --- | --- |
|  | **mtDNA** | | | | **mtDNA+nDNA** | | | |
|  | Gene tree | | Species tree | | Gene tree | | Species tree | |
| Node | Age | 95% HPD | Age | 95% HPD | Age | 95% HPD | Age | 95% HPD |
| n1 | 26.8 | 25.32–29.74 | 26.82 | 24.99–30.12 | 32.88 | 28.75–37.26 | 33.28 | 28.99–37.9 |
| n2 | 19.6 | 18.37–21.89 | 19.51 | 17.53–22.16 | 23.44 | 20.61–26.24 | 23.1 | 20.25–26.01 |
| n3 | 17 | 15.21–19.12 | 16.95 | 15.11–18.32 | 20.28 | 18.38–21.57 | 20.12 | 18.07–21.57 |
| n4 | 8.13 | 6.89–9.31 | 8.33 | 7.09–9.58 | 10.01 | 8.27–11.8 | 9.98 | 8.2–11.79 |
| n5 | 7.37 | 5.92–8.36 | 7.83 | 6.17–8.79 | 8.96 | 7–10.46 | 9.14 | 6.97–10.52 |
| n6 | 4.52 | 3.48–5.62 | 4.64 | 3.52–5.83 | 5.75 | 4.36–7.25 | 5.72 | 4.26–7.26 |
| n7 | 4.42 | 3.5–5.38 | 4.27 | 3.4–5.26 | 5.34 | 4.12–6.62 | 4.93 | 3.77–6.17 |
| n8 | 3.16 | 2.41–3.94 | 3.05 | 2.3–3.34 | 3.85 | 2.89–4.39 | 3.54 | 2.62–4.56 |
| n9 | 2.2 | 1.54–2.88 | 2.13 | 1.42–2.86 | 2.71 | 1.9–3.58 | 2.5 | 1.63–3.38 |

**Table S4**. The independent BioGeoBEARS model test results for the six habitat reconstruction models with consideration of the “founder effect” parameter “J” on geographic regions, biome and topographic barriers datasets. The selected best model of each analysis with its criteria is shown in bold.

|  |  |  |  |  |  |  |  |  |
| --- | --- | --- | --- | --- | --- | --- | --- | --- |
| Geographic |  | log | Number of |  | Parameters |  |  | AICc |
| region | Model | likelihood | parameters | d | e | j | AICc | weight |
|  | DEC | -25.05 | 2 | 0.026 | 0.057 | 0 | 57.1 | 3.60E-02 |
|  | DEC+J | -91.33 | 3 | 0.006 | 3.80E-09 | 0.0034 | 188.8 | 0.039 |
|  | DIVALIKE | -96.35 | 2 | 0.012 | 1.00E-12 | 0 | 196.8 | 0.0007 |
|  | **DIVALIKE + J** | **-88.13** | **3** | **0.007** | **1.00E-12** | **0.0034** | **182.4** | **0.96** |
|  | BAYAREALIKE | -120.8 | 2 | 0.012 | 0.12 | 0 | 245.7 | 1.70E-14 |
|  | BAYAREALIKE + J | -102.8 | 3 | 0.006 | 0.062 | 0.0039 | 211.8 | 3.90E-07 |
|  |  | log | Number of |  | Parameters |  |  | AICc |
| Biome | Model | likelihood | parameters | d | e | j | AICc | weight |
|  | DEC | -25.05 | 2 | 0.026 | 0.057 | 0 | 57.1 | 3.60E-02 |
|  | DEC+J | -91.33 | 3 | 0.006 | 3.80E-09 | 0.0034 | 188.8 | 0.039 |
|  | DIVALIKE | -96.35 | 2 | 0.012 | 1.00E-12 | 0 | 196.8 | 0.0007 |
|  | **DIVALIKE + J** | **-88.13** | **3** | **0.007** | **1.00E-12** | **0.0034** | **182.4** | **0.96** |
|  | BAYAREALIKE | -120.8 | 2 | 0.012 | 0.12 | 0 | 245.7 | 1.70E-14 |
|  | BAYAREALIKE + J | -102.8 | 3 | 0.006 | 0.062 | 0.0039 | 211.8 | 3.90E-07 |
| Topographic |  | log | Number of |  | Parameters |  |  | AICc |
| barrier | Model | likelihood | parameters | d | e | j | AICc | weight |
|  | DEC | -25.05 | 2 | 0.026 | 0.057 | 0 | 57.1 | 3.60E-02 |
|  | DEC+J | -91.33 | 3 | 0.006 | 3.80E-09 | 0.0034 | 188.8 | 0.039 |
|  | DIVALIKE | -96.35 | 2 | 0.012 | 1.00E-12 | 0 | 196.8 | 0.0007 |
|  | **DIVALIKE + J** | **-88.13** | **3** | **0.007** | **1.00E-12** | **0.0034** | **182.4** | **0.96** |
|  | BAYAREALIKE | -120.8 | 2 | 0.012 | 0.12 | 0 | 245.7 | 1.70E-14 |
|  | BAYAREALIKE + J | -102.8 | 3 | 0.006 | 0.062 | 0.0039 | 211.8 | 3.90E-07 |

**Table S5**. The ANOVA based LRT test results retrieved from the likelihood function models of different scenarios investigated under character dependency analyses BiSSE and MuSSE with different models of regions, biomes and the two sides of the Orange River. “Minimal”: the null model assumed all parameters are equal between different characters states; “all different”: the full parameter model considered lambda, mu and q as different between character states; “free.lambda”: the model considered lambda as different only between different character states, “free.mu”: the model considered only mu as different between different character states, “free.lambda.mu”: the model considered only lambda and mu as differ between different character states, “free.q”: the model considered only q as different between different character states. The best model with its criteria from the LRT test in each analysis is shown in bold.

| Object | Model | df | ln Likelihood | AIC | Δ AIC | Chi-square | *p*-value | AIC weight |
| --- | --- | --- | --- | --- | --- | --- | --- | --- |
| Region (MuSSE) | Minimal | 3 | -199.57 | 405.15 | 50.87 | *NA* | *NA* | 8.77E-12 |
|  | all.different | **12** | **-165.14** | **354.28** | **0** | **68.87** | ******* | **9.75E-01** |
|  | free.lambda | 5 | -178.66 | 367.31 | 13.03 | 41.835 | *** | 1.44E-03 |
|  | free.mu | 5 | -176.12 | 362.24 | 7.96 | 46.908 | *** | 1.82E-02 |
|  | free.lambda.mu | 7 | -175.44 | 364.87 | 10.59 | 48.275 | *** | 4.88E-03 |
|  | free.q | 8 | -195.15 | 406.29 | 52.01 | 8.856 | *NS* | 4.95E-12 |
| Biome (MuSSE) | Minimal | 3 | -279.86 | 565.72 | 82.67 | *NA* | *NA* | 1.12E-18 |
|  | all.different | **12** | **-229.52** | **483.05** | **0** | **100.674** | ******* | **1.00E+00** |
|  | free.lambda | 5 | -267.19 | 544.38 | 61.33 | 25.345 | *** | 4.82E-14 |
|  | free.mu | 5 | -264.77 | 539.54 | 56.49 | 30.185 | *** | 5.42E-13 |
|  | free.lambda.mu | 7 | -247.74 | 509.47 | 26.42 | 64.25 | *** | 1.83E-06 |
|  | free.q | 8 | -243.14 | 502.28 | 19.23 | 73.439 | *** | 6.66E-05 |
| Orange River (BiSSE) | all.different | 6 | -157.26 | 326.51 | *NA* | *NA* | *NS* | *NA* |
|  | free.lambda | 5 | **-157.26** | **324.53** | *NA* | 0.018 | *NS* | *NA* |
|  | free.q | 5 | -168.64 | 347.28 | *NA* | 22.769 | *** | *NA* |
| Significance code: | 0–0.001: '***' |  |  |  |  |  |  |  |
|  | 0.001–0.01: '**' |  |  |  |  |  |  |  |
|  | 0.01–0.05: '*' |  |  |  |  |  |  |  |
|  | > 0.05: '*NS*' |  |  |  |  |  |  |  |
|  | “NA”: Not applicable | |  |  |  |  |  |  |
|  | df: degrees of freedom | |  |  |  |  |  |  |

**Table S6**. The average test AUC criterion under the ROC curve and its standard deviation (SD) generated by ENM analyses for each group [*P. t. tentorius* (Ptt-A – Ptt-D), *P. t. trimeni* (Ptr), *P. t. verroxii* (Pv-A and Pv-B), and the entire *P. tentorius* species complex] through the given timeline. LIG: Last Interglacial, LGM: Last Glacial Maximum, and MIDH: Middle Holocene.

|  |  |  |  |  |  |  |  |  |  |  |
| --- | --- | --- | --- | --- | --- | --- | --- | --- | --- | --- |
| Group | **LIG** | | **LGM** | | **MIDH** | | **Current** | | **Future** | |
|  | Test AUC | SD | Test AUC | SD | Test AUC | SD | Test AUC | SD | Test AUC | SD |
| **Ptt-B** | 0.961 | 0.017 | 0.954 | 0.022 | 0.955 | 0.011 | 0.955 | 0.02 | 0.96 | 0.011 |
| **Pv-B** | 0.958 | 0.015 | 0.961 | 0.013 | 0.951 | 0.011 | 0.958 | 0.02 | 0.957 | 0.01 |
| **Ptr** | 0.99 | 0.014 | 0.994 | 0.004 | 0.99 | 0.004 | 0.991 | 0 | 0.992 | 0.005 |
| **Ptt-C** | 0.993 | 0.006 | 0.991 | 0.008 | 0.992 | 0.006 | 0.992 | 0.01 | 0.992 | 0.008 |
| **Ptt-A** | 0.992 | 0.015 | 0.967 | 0.053 | 0.994 | 0.005 | 0.995 | 0 | 0.996 | 0.005 |
| **Pv-A** | 0.977 | 0.034 | 0.973 | 0.043 | 0.967 | 0.045 | 0.963 | 0.05 | 0.973 | 0.039 |
| **Ptt-D** | 1 | 0 | 0.999 | 0.001 | 1 | 0 | 0.995 | 0.01 | 1 | 0 |
| **entire complex** | 0.905 | 0.016 | 0.919 | 0.016 | 0.904 | 0.019 | 0.912 | 0.02 | 0.904 | 0.016 |

**Table S7**. The results of the optimization of parameters settings for all ENM analyses across groups (group abbreviations as in Table S6, with All denoting the entire *P. tentorius* species complex) and timelines. Features: Linear features (L), Quadratic features (Q), Product features (P) and Hinge features (H); RM = Regularization multiplier. LIG: Last Interglacial, LGM: Last Glacial Maximum, and MIDH: Middle Holocene.

| Group | **LIG** | | **LGM** | | **MIDH** | | **Current** | | **Future** | |
| --- | --- | --- | --- | --- | --- | --- | --- | --- | --- | --- |
|  | Features | RM | Features | RM | Features | RM | Features | RM | Features | RM |
| **Ptt-B** | LQ | 0.5 | LQ | 0.5 | LQ | 0.5 | LQH | 3 | LQH | 2.5 |
| **Pv-B** | LQHP | 1.5 | LQHP | 2 | LQ | 0.5 | LQH | 2 | LQH | 1.5 |
| **Ptr** | LQH | 3 | LQ | 0.5 | LQ | 0.5 | LQ | 1 | LQ | 0.5 |
| **Ptt-C** | LQH | 2.5 | LQ | 2.5 | LQHP | 2.5 | LQHP | 1.5 | LQHP | 1.5 |
| **Ptt-A** | LQHP | 1 | LQHP | 1 | LQHP | 1 | LQHP | 1 | LQHP | 1 |
| **Pv-A** | LQH | 3 | LQH | 1.5 | LQ | 0.5 | LQ | 0.5 | LQH | 2.5 |
| **Ptt-D** | LQHP | 0.5 | LQHP | 0.5 | LQHP | 0.5 | LQHP | 0.5 | LQHP | 0.5 |
| **All** | LQH | 2 | LQ | 0.5 | LQHP | 1 | LQHP | 1 | LQHP | 2.5 |

**Table S8**. The pairwise results of niche difference analysis between clades (taxon abbreviations the same as group abbreviations in Table S6). The pairwise comparisons showing significant niche differences are in bold, whilst, comparisons without significant niche differences are underlined. Note: If the point estimated value (the matrix at the bottom-left) was lower than the 5% threshold value (the matrix at the top-right), then the niches were significantly different.

|  |  |  |  |  |  |  |  |
| --- | --- | --- | --- | --- | --- | --- | --- |
| Taxon | Ptt-B | Pv-B | Ptr | Ptt-C | Ptt-A | Pv-A | Ptt-D |
| Ptt-B | 1.00 | 0.79 | 0.75 | 0.79 | 0.59 | 0.70 | 0.64 |
| Pv-B | **0.78** | 1.00 | 0.74 | 0.76 | 0.69 | 0.75 | 0.72 |
| Ptr | **0.39** | **0.19** | 1.00 | 0.80 | 0.72 | 0.71 | 0.72 |
| Ptt-C | **0.65** | **0.56** | **0.48** | 1.00 | 0.75 | 0.76 | 0.72 |
| Ptt-A | 0.67 | **0.38** | **0.55** | **0.67** | 1.00 | 0.66 | 0.87 |
| Pv-A | **0.44** | **0.55** | **0.09** | **0.25** | **0.20** | 1.00 | 0.70 |
| Ptt-D | 0.81 | **0.55** | **0.45** | **0.65** | **0.66** | **0.21** | 1.00 |
|  |  |  |  |  |  |  |  |

**Table S9**. The ENM analyses estimating the area of suitable habitats of the seven clades (group abbreviations as in Table S6), as well as changes in the area of suitable habitats against the timeline (for details about the timeline see the Materials and Methods). The arrow " ↑ " represents increasing area, whilst, " ↓ " indicates decreasing area. Groups currently showing a decrease in the area of suitable habitats or future habitats are given in bold.

|  |  |  |  |  |  |  |  |  |  |
| --- | --- | --- | --- | --- | --- | --- | --- | --- | --- |
|  | **LIG** | **LGM** |  | **MIDH** |  | **Current** |  | **Future (km²)** |  |
| Ptt-B | 309455.59 | 367477.15 | **↑** | 322701.53 | ↓ | 361375.33 | ↑ | 216495.14 | **↓** |
| Change |  | 58021.57 |  | -44775.62 |  | 38673.81 |  | **-144880.20** |  |
| Pv-B | 289803.82 | 297078.23 | ↑ | 305394.95 | ↑ | 297729.67 | **↓** | 271476.65 | **↓** |
| Change |  | 7274.41 |  | 8316.71 |  | **-7665.27** |  | **-26253.02** |  |
| Ptr | 69161.18 | 63819.38 | ↓ | 62386.21 | ↓ | 78498.49 | ↑ | 67380.58 | **↓** |
| Change |  | -5341.81 |  | -1433.17 |  | 16112.28 |  | **-11117.90** |  |
| Ptt-C | 76088.16 | 67597.73 | ↓ | 69769.20 | ↑ | 61105.05 | **↓** | 66555.43 | ↑ |
| Change |  | -8490.43 |  | 2171.47 |  | **-8664.15** |  | 5450.38 |  |
| Ptt-A | 98649.69 | 118410.03 | ↑ | 151894.03 | ↑ | 137584.07 | **↓** | 123404.40 | **↓** |
| Change |  | 19760.34 |  | 33484.00 |  | **-14309.96** |  | **-14179.67** |  |
| Pv-A | 276036.73 | 229784.51 | ↓ | 226918.17 | ↓ | 263029.65 | ↑ | 235517.18 | **↓** |
| Change |  | -46252.22 |  | -2866.33 |  | 36111.48 |  | **-27512.47** |  |
| Ptt-D | 17241.44 | 13463.09 | ↓ | 8794.44 | ↓ | 72070.95 | ↑ | 80648.24 | ↑ |
| Change |  | -3778.35 |  | -4668.65 |  | 63276.51 |  | 8577.29 |  |
| All | 640734.41 | 614264.24 | ↓ | 572246.38 | ↓ | 522997.53 | **↓** | 553137.48 | ↑ |
| Change |  | -26470.17 |  | -42017.86 |  | **-49248.84** |  | 30139.94 |  |
|  |  |  |  |  |  |  |  |  |  |

**Table** **S10***. The climatic variables that made significant impacts in each group across different periods (group abbreviations as in Table S6) from the ENM analyses.

**Table S11***. List of all samples, their corresponding localities and NCBI GenBank accession numbers across different genes. All NCBI accessions of outgroups used in this study are given at the bottom.

**Table S12***. List of primers used in the study with corresponding oligo sequences, optimized annealing temperatures and sources.

**Table S13**. Optimal partition scheme, substitution model, likelihood score (-InL), Gamma shape, proportion of estimated invariable sites for the mtDNA+nDNA based BEAST phylogenetic inference.

|  |  |  |  |  |
| --- | --- | --- | --- | --- |
| Partition scheme | Model | -InL | Gamma shape | P-inv |
| *12S* | TIM3+I+G | 1733.56 | 0.661 | 0.397 |
| *16S* | TIM2+I+G | 2096.84 | 0.58 | 0.511 |
| *Cytb_*1 | TPM2uf+I | 1090.84 | NA | 0.6 |
| *ND4_*2*, Cytb_*2 | TPM3uf+G | 648.6 | 0.1 | NA |
| *Cytb_*3 | TVM+I+G | 2250.85 | 4.99 | 0.03 |
| *ND4_*1 | TrN+G | 1254.58 | 0.306 | NA |
| *ND4_*3 | TIM1+G | 2303.49 | 2.41 | NA |
| *tRNA* | TrN+G | 448.23 | 0.35 | NA |
| *PRLR_*1 | TPM1uf+I | 338.38 | NA | 0.79 |
| *PRLR_*2 | TrN+G | 379.73 | 0.45 | NA |
| *PRLR_*3 | K80 | 438.53 | NA | NA |
| Note: " * " - *p* < 0.05 | |  |  |  |
| " ** " - *p* < 0.01 | |  |  |  |
| " *** " indicate *p* < 0.0001 | |  |  |  |
| "NA"- Not applicable | |  |  |  |

**Table S14***. Allele size ranges, repeat motifs with their NCBI GenBank accession numbers, multiple-mix reaction grouping schemes, the oligo-nucleotide sequences of primers with dyes, optimal annealing temperatures and the sources of primers of all microsatellite DNA markers tested in this study. "NA" indicates the primer pair failed to amplify.

**Table S15***. The genotyping results of 14 microsatellite DNA loci used in this study, together with locality and subpopulation information.

**Table S16**. Optimal partition scheme, substitution model, likelihood score (-InL), Gamma shap, and proportion of estimated invariant sites for the BEAST calibration dating analyses.

|  |  |  |  |  |
| --- | --- | --- | --- | --- |
| Partition scheme | Model | -InL | Gamma shape | P-inv |
| *12S* | GTR+I+G | 1733.3 | 0.682 | 0.407 |
| *16S* | GTR+I+G | 2078.7 | 0.598 | 0.513 |
| *Cytb_*1 | TVM+I+G | 1111.64 | 0.677 | 0.589 |
| *Cytb_2，ND4_2* | GTR+I+G | 650.18 | 0.461 | 0.462 |
| *Cytb_*3 | GTR+I+G | 2257.6 | 5.368 | 0.044 |
| *ND4_*1 | TrN+G | 1244.97 | 0.309 | NA |
| *ND4_*3 | TIM1+G | 2327.01 | 2.193 | NA |
| *tRNA* | TrN+G | 447.4 | 0.41 | NA |
| *PRLR_*1 | HKY+I | 318.72 | NA | 0.496 |
| *PRLR_*2 | TrN | 361.84 | NA | NA |
| *PRLR_*3 | HKY+I+G | 427.63 | 1.63 | 0.288 |
| Note: " * " - *p* < 0.05 | |  |  |  |
| " ** " - *p* < 0.01 | |  |  |  |
| " *** " indicate *p* < 0.0001 | |  |  |  |
| "NA"- Not applicable | |  |  |  |

**Table S17**. The constraint of the five most recent common ancestors used in the BEAST calibration dating analyses for the mtDNA and mtDNA+nDNA datasets.

|  |  |  |  |
| --- | --- | --- | --- |
| MRCA | Node position | mtDNA (Ma) | mtDNA+nDNA (Ma) |
| 1 | *P. oculifer*+*P. geometricus* | 16.89 | 16.89–21.57 |
| 2 | *Stigmochelys*+*Psammobates* | 26.63 | 26.63–39.72 |
| 3 | *Chersina*+*Chersobius* | 19.68 | 19.68–29.51 |
| 4 | *Homopus*+(*Chersina*+*Chersobius*) | 28.64 | 28.64–42.20 |
| 5 | *Astrochelys*+*Pyxis* | 26.44 | 26.44–36.29 |

**Table S18***. The bioclimatic variables and their percentage contribution, permutation importance and jack-knife test AUC’s used in the ENM to determine suitable habitat ranges of the *P. tentorius* species complex. Variables with AUC above 0.75 were considered as potentially useful (given in bold).

**Table S19***. The correlation matrix retrieved from the correlation test between each pair of bioclimatic variables. The R-square values varied from zero to one, with "zero" representing no correlation and "one" representing 100% correlation. The bioclimatic variables (in bold) are the ones used in further niche modelling analyses after trimming variables showing strong correlation. The pairwise values (R-square) > 70 or < -70 were considered as strong correlations. All pairwise R-square values showing a strong correlation between bioclimatic variables are underlined.

**2. Supplementary Figures**


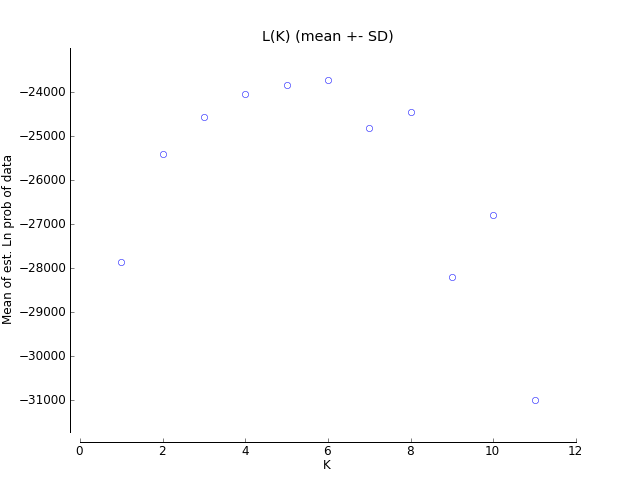


**Figure S1**. The results of the Bayesian clustering analysis with STRUCTURE. The plot shows the values of *ΔK* calculated according to Evanno et al*.* [47].


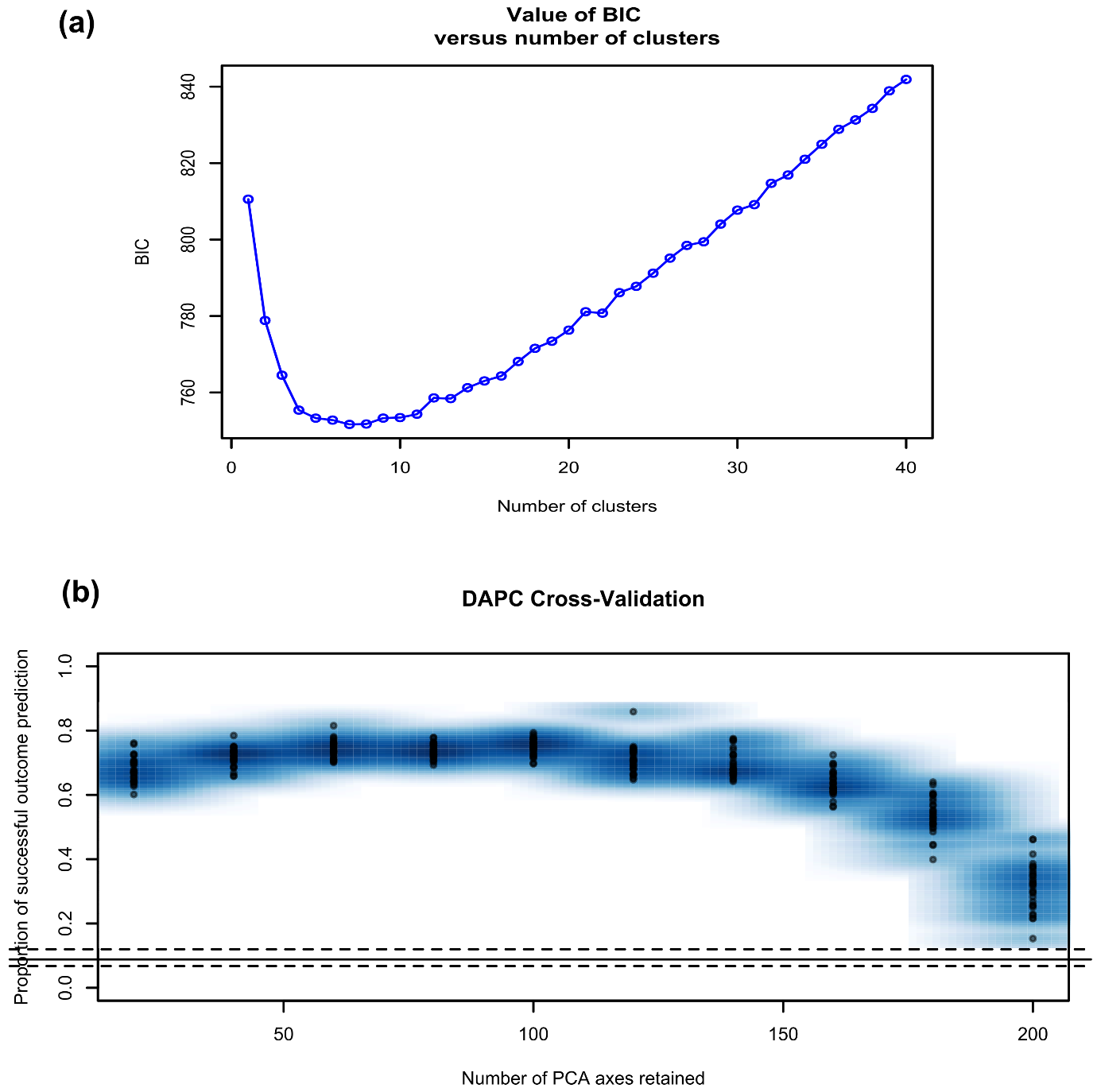


**Figure S2**. (a) Results of BIC value versus the number of clusters to determine the optimal clustering scheme in the DAPC analyses. (b) The Cross-Validation test results determining the optimal number of PCs retained.


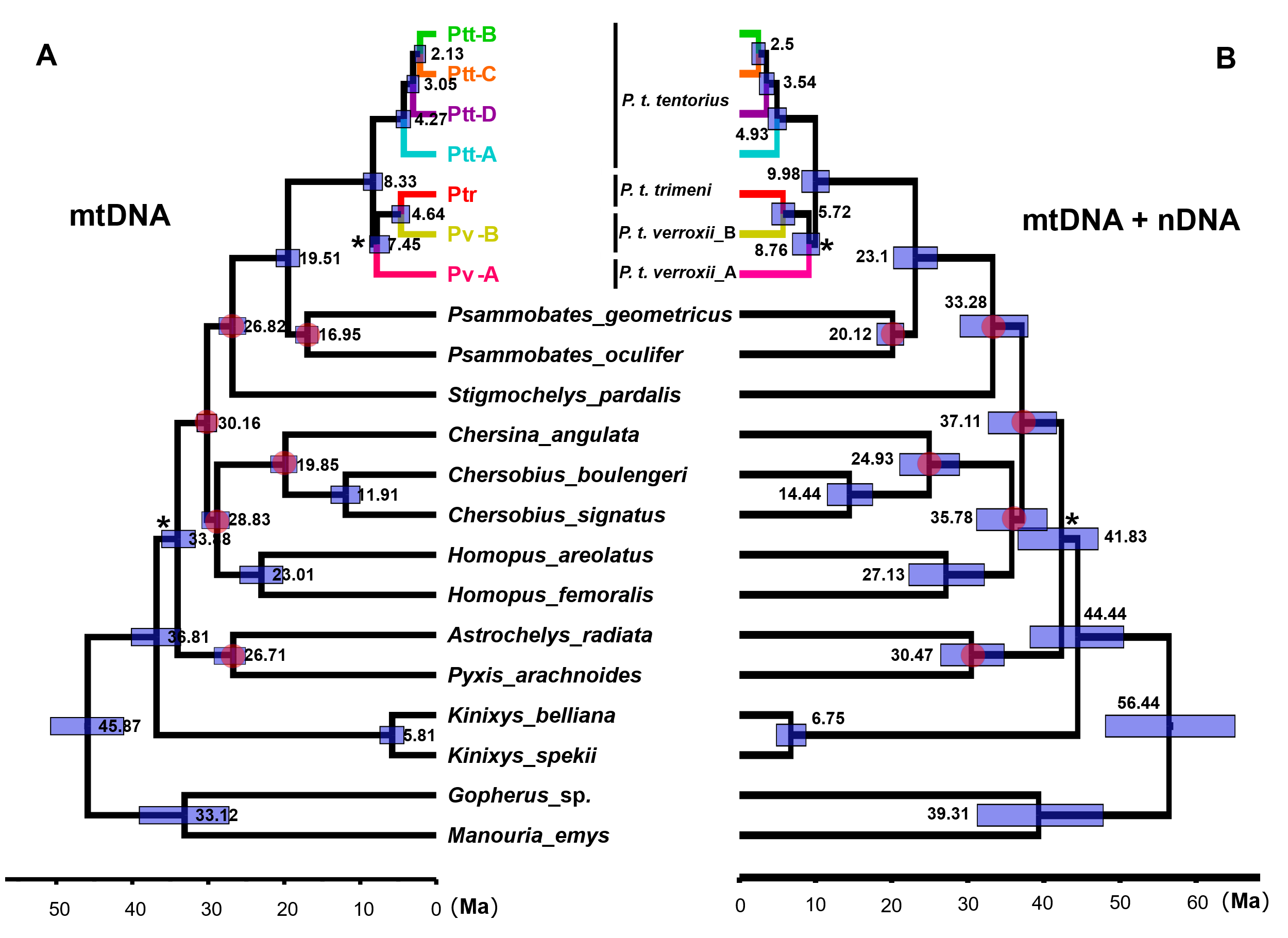


**Figure S3**. The species tree chronograms generated from the BEAST calibration dating analyses, A: mtDNA chronogram, B: mtDNA+nDNA chronogram. The red dots were the five constrained calibration points for calibration dating analyses.


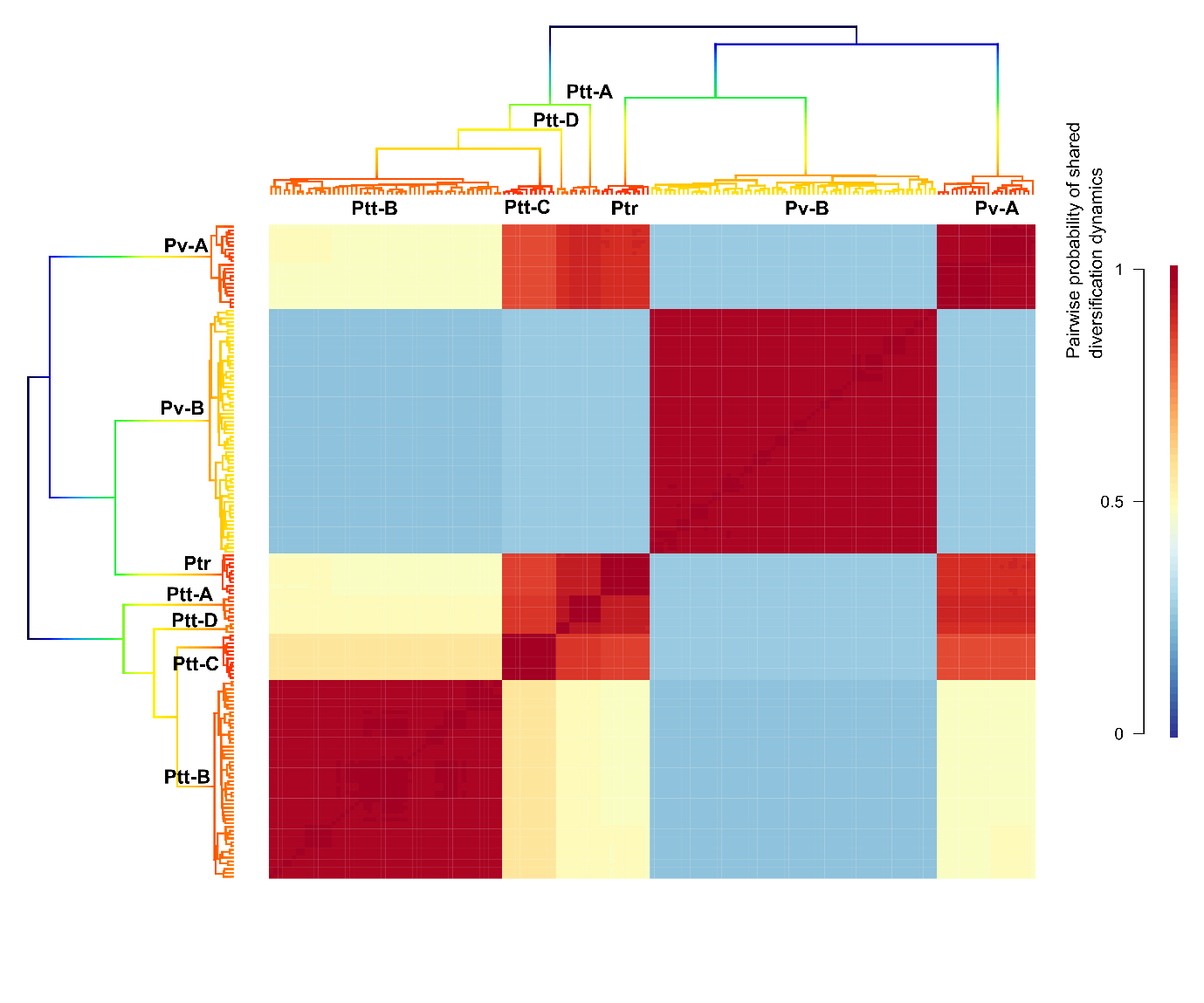


**Figure S4**. The macroevolution cohort matrix for the seven clades of the *P. tentorius* species complex. BAMM Bayesian diversification rate analysis based on the mean phylorate plot trees, are shown at the top and on the left side of the cohort matrix, for purposes of comparison. The matrix shows pairwise probabilities of two groups sharing the same evolutionary dynamics. The “warm” colours represent high cohort similarities (highest value “1” refers to 100% similarity), whilst, the “cool” colours represent low cohort similarities (lowest value “0” refers to 0% similarity).


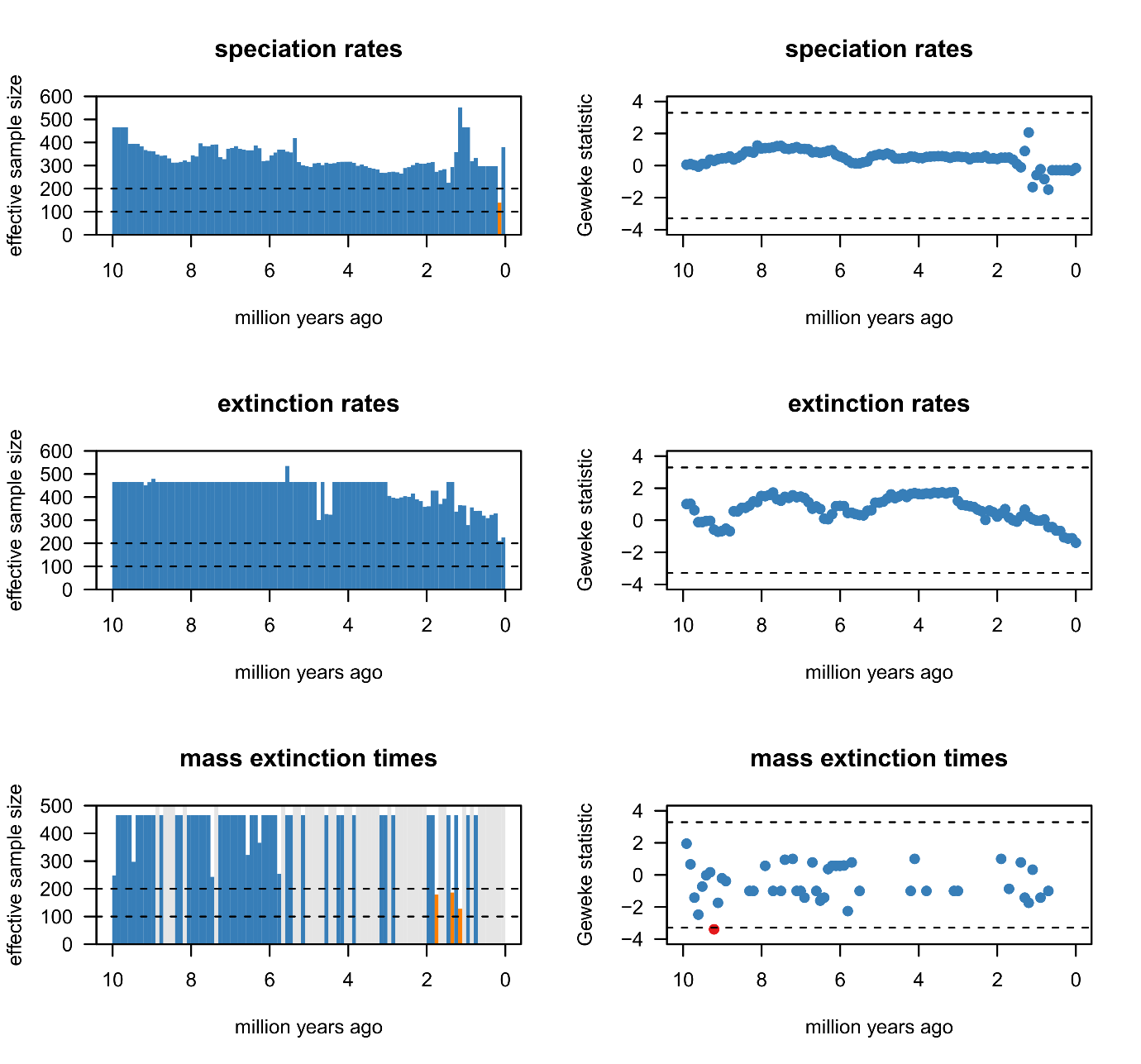


**Figure S5**. Visualizing the single-chain MCMC diagnostics for a CoMET analysis with empirically estimated diversification hyperpriors. Blue bars/dots represent passed tests and red bars/dots mean failed tests (failed convergence).

**References**

[1] Zhao Z, Heideman N, Grobler P, Jordaan A, Bester P, Hofmeyr MD. Unraveling the diversification and systematic puzzle of the highly polymorphic *Psammobates tentorius* (Bell, 1828) complex (Reptilia: Testudinidae) through phylogenetic analyses and species delimitation approaches. J Zool Syst Evol Res. 2020;58:308–26. https://doi.org/10.1111/jzs.12338.
